# Heat dissipation analysis and multi-objective optimization of microchannel liquid cooled plate lithium battery pack

**DOI:** 10.1371/journal.pone.0313594

**Published:** 2024-12-05

**Authors:** Xueyong Pan, Chuntian Xu, Xuemei Sun, Jianhui Shi, Zhilong Zhou, Yunlong Liu

**Affiliations:** 1 School of Mechanical & Vehicle Engineering, Linyi University, Shandong, China; 2 School of Mechanical Engineering & Automation, Liaoning Science and Technology University, Liaoning, China; Birla Institute of Technology and Science Pilani - K K Birla Goa Campus, INDIA

## Abstract

An efficient battery pack-level thermal management system was crucial to ensuring the safe driving of electric vehicles. To address the challenges posed by insufficient heat dissipation in traditional liquid cooled plate battery packs and the associated high system energy consumption. This study proposes three distinct channel liquid cooling systems for square battery modules, and compares and analyzes their heat dissipation performance to ensure battery safety during high-rate discharge. The results demonstrated that the extruded multi-channel liquid cooled plate exhibits the highest heat dissipation efficiency. Subsequently, response surface experiments were conducted to analyze the width parameters of various flow channels in the liquid cooled plate Finally, the Design of Experiment (DOE) was employed to conduct optimal Latin hypercube sampling on the flow channel depth (*H*), mass flow (*Q*), and inlet and outlet diameter (*d*), combined with a genetic algorithm for multi-objective analysis. The *T*_max_ of the battery module decreased by 6.84% from 40.94°C to 38.14°C and temperature mean square deviation decreased (*TSD*) by 62.13% from 1.69 to 0.64. Importantly, the battery thermal management model developed in this study successfully met heat dissipation requirements without significantly increasing pump energy consumption.

## 1. Introduction

New energy vehicles have attracted considerable attention worldwide due to their environmentally friendly and sustainable characteristics [1]. The primary power source for new energy vehicles is the power battery, whose performance directly impacts both the vehicle’s maneuverability and safety. Currently, the primary types of power batteries include nickel-hydrogen batteries, fuel cells, and lithium-ion batteries (LIBs). LIBs have various advantages in practical applications [2–4], including high energy density, high power factor, long cycle life, low self-discharge rate, good stability, and no memory effect. Therefore, they are widely used as power batteries for new energy vehicles. The successful development of electric vehicles powered by lithium-ion batteries has significantly propelled the advancement of new energy electric vehicles. Nevertheless, the chemical reactions occurring within LIBs during the charging and discharging processes generate substantial heat, leading to rapid temperature increases, which accelerate battery aging and affect its service life. In severe cases, the lithium-ion battery can experience thermal runaway [5], resulting in issues such as spontaneous combustion and explosion [6]. Due to the characteristics of lithium-ion batteries, the allowable working temperature should be controlled within 20~40°C, with a maximum temperature difference not exceeding 5°C [7]. To ensure the safety of electric vehicles, it is crucial to control the battery temperature within the optimal range during operation, necessitating comprehensive research into the Battery Thermal Management System (BTMS) [8, 9].

The BTMS utilized temperature monitoring and integrated multidisciplinary knowledge, including heat transfer, hydrodynamics, and material science. It realized temperature control of the entire and individual battery pack to ensure they operated within a safe range and maximized their performance [10]. Currently, the heat dissipation methods for battery packs include air cooling [11], liquid cooling [12], phase change material cooling [13], heat pipe cooling [14], and popular coupling cooling [15]. Among these methods, due to its high efficiency and low cost, liquid cooling was widely used by most enterprises. Yao et al. [16] designed a biomimetic spider web channel and analyzed four different cooling channel schemes. The results indicate that scheme (b) exhibits the best heat dissipation performance. Zhang et al. [17] designed a new type of biomimetic fin vein flow channel cold plate and conducted a comparative analysis of heat dissipation performance with two parallel flow channel cold plates with different inlet and outlet positions. It was found that the new biomimetic fin vein flow channel cold plate can not only further reduce the maximum temperature and temperature difference but also improve temperature uniformity, while also reducing pressure drop and greatly reducing energy loss. Zhang et al. [18] investigated the impact of a novel special-shaped needle fin on liquid flow and heat dissipation within the channel. The structure was found to disturb the flow of the coolant, which leads to an increase in the flow rate nearby, thereby improving the overall heat dissipation performance. This implies that the design of a special-shaped needle fin can effectively improve the heat transfer efficiency of an LCP. Luo et al. [19] designed a square spiral annular LCP, and its effects on heat dissipation and pressure drop of the battery were investigated by varying parameters such as the number of flow channels, width, corner radius, and inlet coolant speed. Feng et al. [20] analyzed the material of the LCP, the structure of the channel, and the flow rate of the coolant using the entropy weight method of information theory. The study revealed that the channel structure significantly impacts both heat dissipation performance and temperature uniformity. Aida Salimi et al. [21] studied a novel wavy microchannel cold plate. By analyzing five different wave amplitudes of wave microchannels and analyzing the heat dissipation effects in both the co-flow and counter-flow modes, it was found that the overall heat dissipation performance of the cold plate in the counter-flow mode was better. Elham Hosseinirad et al. [22] evaluated the performance of miniature heat sinks by combining straight fins and wavy fins and tested different interruption models to determine the optimal solution. Research has shown that the proximity between straight and wavy fins can affect the thermal and hydraulic performance of miniature heat sinks. Zhao et al. [10] investigated how the number of channels in a liquid-cooled plate affects battery pack heat dissipation and found that a single-channel plate performs best. On this basis, the channel width, height, and coolant flow rate were optimized through orthogonal experiments. Adding another liquid-cooled plate above the battery pack reduced *T*_*max*_ to 27.7°C and *ΔT*_*max*_ to 1.9°C. Chen et al. [23] proposed a parallel liquid cooling system, Sensitivity analysis and response surface analysis were conducted on the structural parameters of the liquid-cooled plate, and the influence of each parameter on the heat dissipation of the battery pack was investigated. Subsequently, through multi-objective optimization design, the *T*_*max*_, *TSD*, and required power of the battery module were reduced to 33.1°C, 0.9°C, and 17.29 J, respectively.

The above mainly involves changing the shape of its liquid cooling channel and adding disturbance elements to the liquid cooling channel, in order to analyze the distribution of coolant and the impact of flow rate on the heat dissipation of battery cells. Most studies only focus on a single battery or a few batteries, with relatively little research on multiple battery packs. In this study, three different liquid-cooled plate channels were proposed, and the optimal structure was obtained by comparing and analyzing the temperature distribution and required power of the battery pack during discharge. Based on this, conduct Box-Behnken experimental design on the channel width parameters to find the optimal parameters. Finally, a multi-objective analysis is conducted on the channel depth, coolant mass flow rate, and inlet and outlet diameters to find the optimal cooling conditions.

## 2. Mathematic model

### 2.1. Control equation

The heat transfer between the battery and the liquid cooled plate mainly relies on thermal conduction. Heat is transferred from the battery to the liquid cooling plate through the thermal conductivity of solid materials and then carried away by the coolant on the liquid cooling plate. Drawing upon the investigation by Chen et al. [24], the phenomenon can be mathematically formulated through the utilization of the Fourier three-dimensional heat conduction differential equation:

$$\rho C\frac{\partial T}{\partial t}=\frac{\partial }{\partial x}(\lambda_{x}\frac{\partial T}{\partial x})+\frac{\partial }{\partial y}(\lambda_{y}\frac{\partial T}{\partial y})+\frac{\partial }{\partial z}(\lambda_{z}\frac{\partial T}{\partial z})+q$$ (1)

where *ρ* is the average physical density, kg/m^3^; *C* is the average specific heat capacity, J/(kg k); *λ*_*x*,*y*,*z*_ is the heat coefficient of each guide along the coordinate axis, W/(m·K); *q* is the heat production rate of the internal heat source of the battery, W/m^3^.

The momentum equation, continuity equation and energy conservation equation of the coolant are as follows [25]:

$$\rho_{w}[\frac{\partial \vec{\nu }}{\partial t}+(\vec{\nu }\cdot \nabla )\vec{\nu }]=-\nabla P+\mu \nabla^{2}\vec{\nu }$$ (2)

$$\nabla \cdot \vec{\nu }=0$$ (3)

$$\rho_{w}C_{w}\frac{\partial T_{w}}{\partial t}+\nabla \cdot (\rho_{w}C_{w}\vec{\nu }T_{w})=\nabla \cdot (\lambda_{w}\nabla T_{w})$$ (4)

where $\vec{\nu }$ is the vector velocity of the coolant, m/s; *ρ*_*w*_ is the density of the coolant, kg/m^3^; *P* is the static pressure of the coolant, pa; *μ* is the dynamic viscosity of the coolant, pa·s; *C*_*w*_ is the specific heat capacity of the coolant, J/(kg·K); *λ*_*w*_ is the thermal conductivity of the coolant, W/(m·K); *T*_*w*_ is the temperature of the coolant, K; ∇ is the Hamiltonian operator.

Energy conservation equation of liquid-cooled plate [26]:

$$\rho_{l}C_{l}\frac{\partial T_{l}}{\partial t}=\lambda_{l}\nabla^{2}T_{l}$$ (5)

where *ρ*_*l*_ is the density of the LCP, kg/m^3^; *C*_*l*_ is the specific heat capacity of the LCP, J/(kg·K); *λ*_*l*_ is the thermal conductivity of the LCP, W/(m·K); *T*_*l*_ is the temperature of the LCP, K.

### 2.2. Calculation of heat source

Numerous scholars, both domestic and international, have proposed diverse models for battery heat generation. Notably, the model formulated by Bernardi et al. [27] has gained widespread recognition and adoption. The specific formula of the heat generation model is as follows:

$$q=\frac{1}{V}[(U_{0}-U)-T\frac{\partial U_{0}}{\partial T}]=\frac{1}{V}(I^{2}R-IT\frac{\partial U_{0}}{\partial T})$$ (6)

where *q* is the heat generation rate of lithium-ion battery, W/m^3^; *I* is the charge and discharge current, A; *U*_*0*_ is the open-circuit voltage of the battery, V; *U* is the working voltage of the battery, V; *T* is the working temperature of the battery, K; *R* is the internal resistance of the battery, mΩ; *V* is the volume of the battery, m^3^; ∂*U*_0_/∂*T* is the temperature influence coefficient, which is determined by the chemical reaction of the lithium battery.

**Fig 1:**
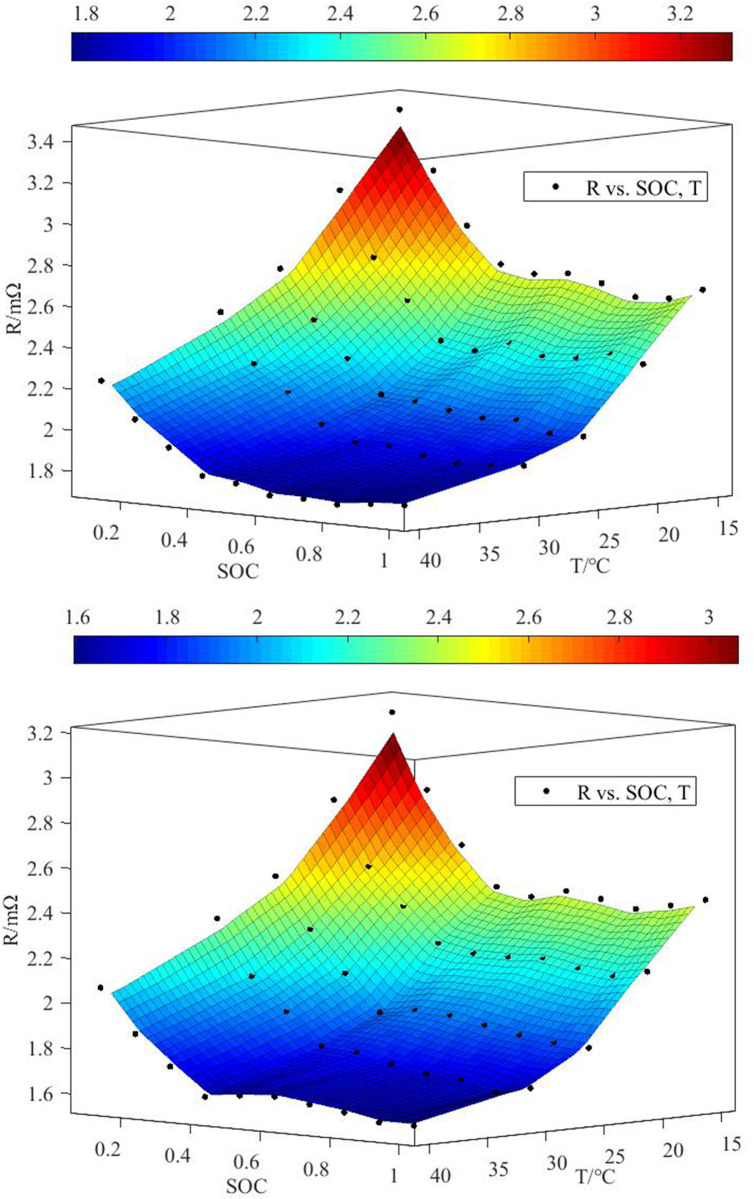
The relationship between *R* and different discharge rates under different *T* and *SOC*. (a) Battery discharge rate 1C, (b) Battery discharge rate 2C.


Based on experimental data presented in reference [28], as shown in Fig 1, the internal resistance of a battery was not constant, and it was mainly affected by ambient temperature, battery state of charge (*SOC*), and discharge rate. With increasing temperature, the internal resistance of the battery gradually decreases, and when the *SOC* was below 20%, the internal resistance of the battery increases sharply. The temperature coefficient ∂*U*_*0*_*/*∂*T* can be regarded as a constant value. Because the study focused on the lithium-ion battery’s best working range (25°C~40°C), the internal resistance and *SOC* value of the battery were less affected by temperature changes when the battery operated within the range of 25°C to 40°C. To minimize the amount of fitting calculation, the effect of temperature on *SOC*, as described in Eq (7), could be ignored. Upon consulting the relevant literature [29], it was determined that the temperature influence factor is 0.469 mv/°C. The study utilized an expression function to establish a polynomial relationship between heating rate and *SOC*, in order to define the battery’s heat source. Following this, Formulas (7) and (8) were to be substituted into (6).

$$R^{*}=\{\begin{array}{l} R_{1C}=2.86-0.99*SOC-10.18*SOC^{2}+\\ 32.60*SOC^{3}-35.22*SOC^{4}+12.95*SOC^{5}\\ R_{2C}=2.63-0.47*SOC-13.99*SOC^{2}+\\ 44.05*SOC^{3}-48.95*SOC^{4}+18.59*SOC^{5} \end{array}$$ (7)

*SOC* is an indicator for measuring remaining electricity. It has a value of 1 when fully charged, and a value of 0 when completely discharged. The *SOC* value estimation method proposed by Cheng [30] is presented in Formula (8):

$$SOC=SOC_{Init}-\frac{1}{C_{N}}\int_{0}^{t}Idt$$ (8)

where *SOC*_*Init*_ represents the starting power value, *C*_*N*_ is the rated capacity of the battery, and *t* is the charging (discharging) time of the battery.

### 2.3. Reynolds number

The Reynolds number (*Re*) is the relative ratio of inertial and viscous forces in describing fluid flow, which can determine the type of fluid flow. The equation is expressed as follows [31]:

$$Re=\frac{\rho vDe}{\mu }$$ (9)

where *De* represents the entrance diameter of the channel. When the *Re* is less than 2300, it is a laminar flow model; When the *Re* is between 2300 and 13800 inclusive, the model can choose between laminar flow or turbulence; Finally, when the *Re* surpasses 13800, it is a turbulence model. In this study, the liquid mass flow is 11.29 g∙s^-1^, and the *Re* is 1198, which is smaller than the laminar *Re*, it can be concluded that the model exhibits laminar flow under this condition. Subsequently, the value of *Re* is calculated based on the different flow speeds of the coolant to determine the type of coolant flow.

### 2.4. Hypothesis and establishment of simulation model of LIBs

Due to the complexity of the battery’s interior and the variability of its chemical reactions, it is impossible to directly establish an accurate three-dimensional thermal effect model. Therefore, it becomes essential to simplify and assume the battery model:

- The internal materials of the battery are uniformly distributed, and the thermal conductivity remains consistent in the same direction, maintaining anisotropy;
- The battery’s thermophysical parameters remain constant, regardless of temperature or charge state;
- Neglecting the radiation and thermal deformation inside the battery, as well as ignoring the positive and negative electrode;
- Ignore the thermal contact resistance between the surfaces.

The object of the study is a square battery. The battery specifications are shown in Table 1, and the parameters for the three-dimensional heat transfer model are shown in Table 2.

**Table 1:** Related parameters of square LIBs.

Technical parameters	Parameter value
Rated capacity	40Ah
Rated voltage	3.7V
Charging cut-off voltage	4.2V
Discharge cut-off voltage	2.8V
Charging temperature	0~45°C
Battery size	148*27*91mm

**Table 2:** Three-dimensional heat transfer model parameters.

Parameter	*ρ*(kg/m^3^)	*C*(J/kg∙K)	*λ*(W/m∙K)	*μ*(pa∙s)
LIBs	2218	1033	17.4/5.3/23	\
Water	998.2	4182	0.6	0.001003
Aluminum alloy Silicone pad	2702 2000	903 1800	237 1.8	\ **\**
Epoxy plate	1800	1581	0.2	\

### 2.5. Experimental verification

**Fig 2:**
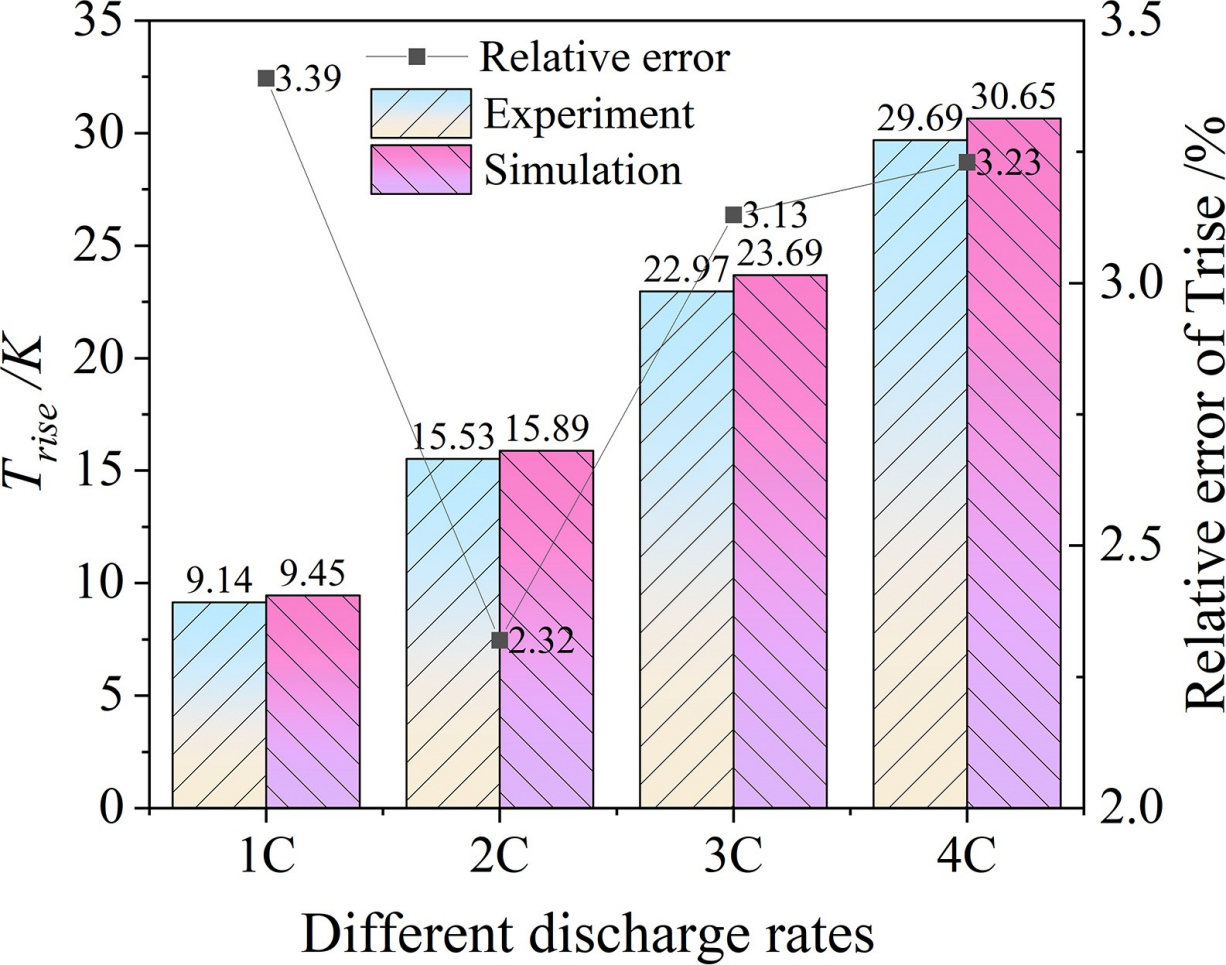
Experiment and simulation of discharge at different discharge rates.

To ensure simulation feasibility, an experimental analysis was conducted on a specific type of battery’s single discharge model. The experimental verification was based on the experimental data provided by Li et al. [32] in the literature. The experiment and simulation of single cell battery discharge at different discharge rates as shown in Fig 2. By comparing the experimental data *T*_*rise*_ (the temperature difference between the maximum and initial values.) with simulation data, it could be seen that when the discharge rates were 1C, 2C, 3C, and 4C, the relative error of the *T*_*rise*_ was also within 3.5%. There was no significant difference between the experimental data and the simulation data, indicating that the simulation data was accurate, reliable, and could be further utilized in the current research.

### 2.6. Grid independence verification

**Fig 3:**
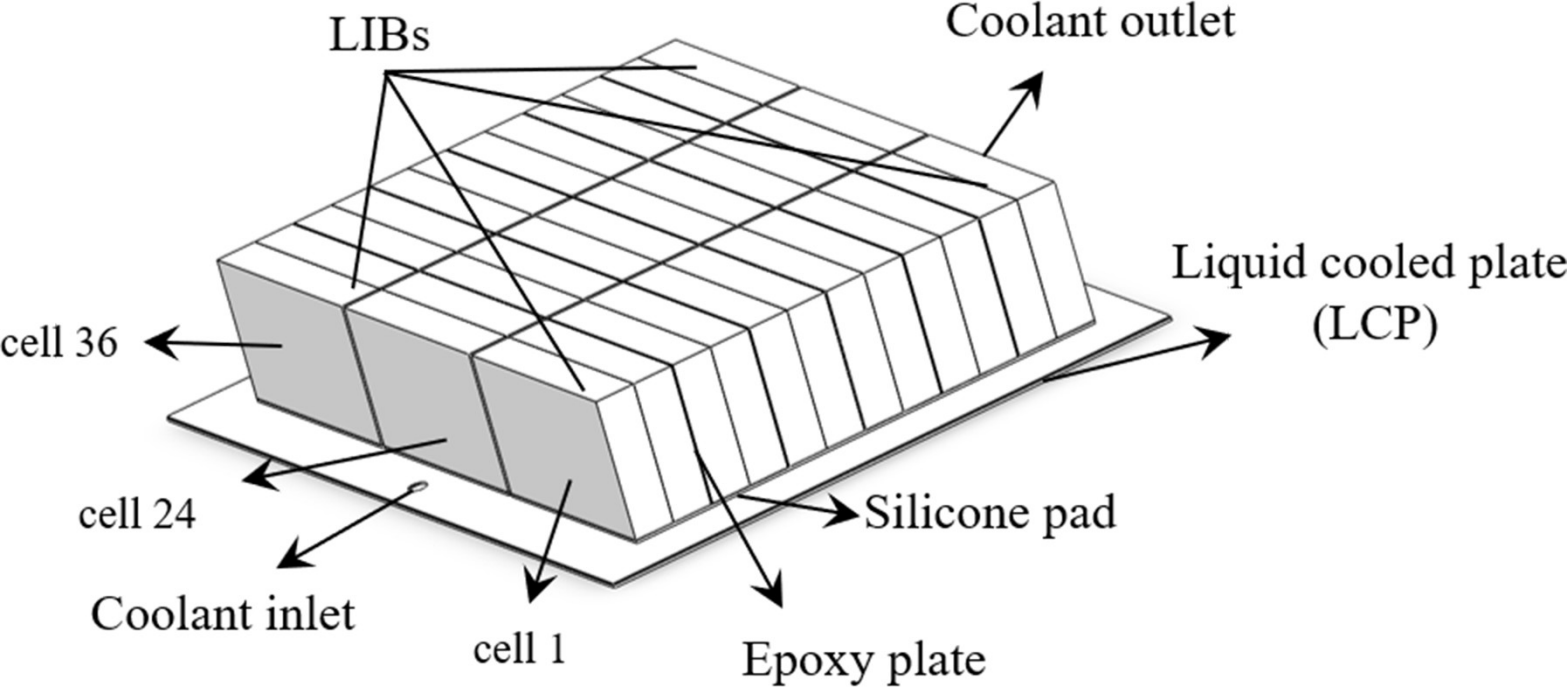
Battery pack model.

**Fig 4:**
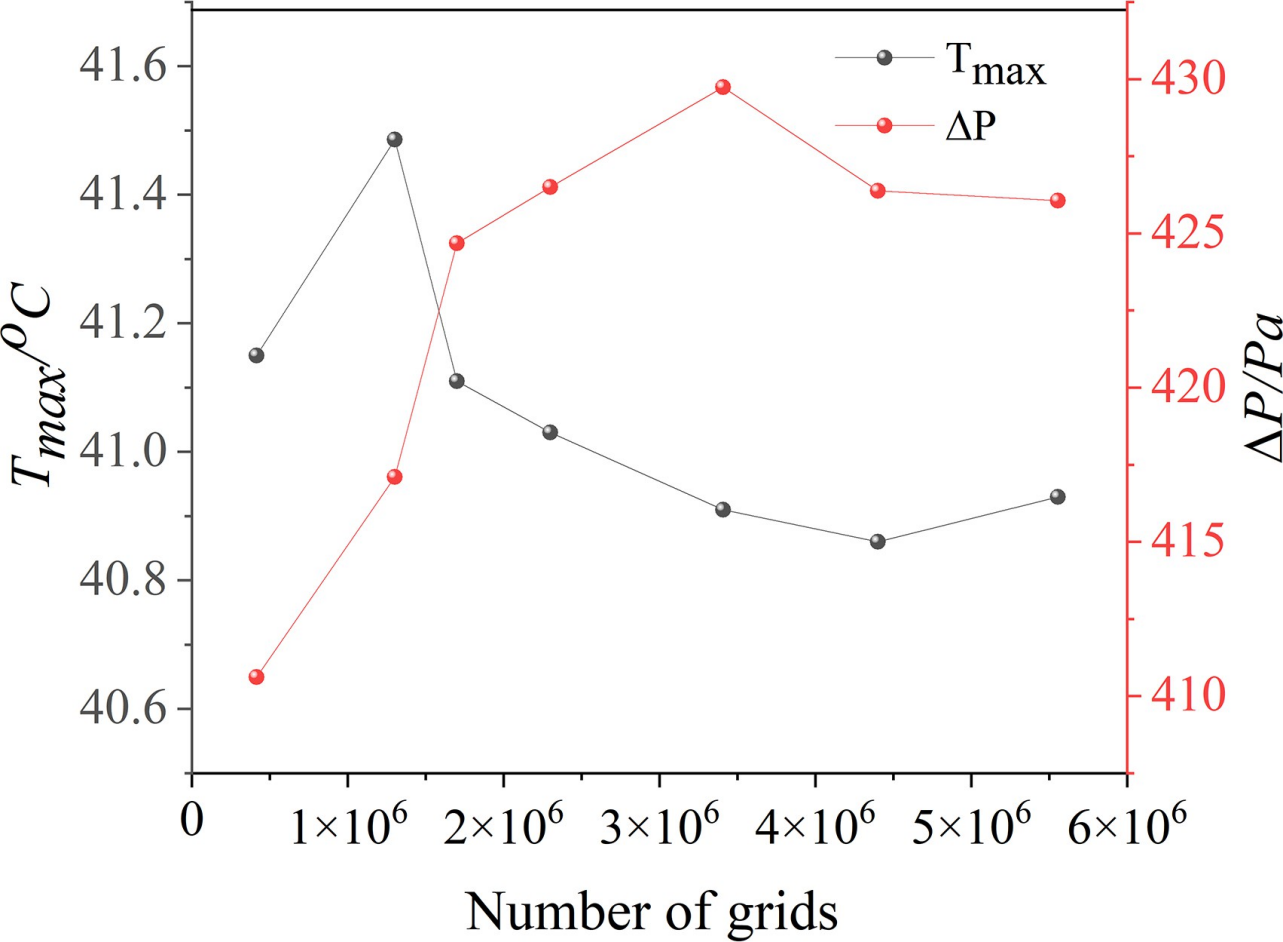
2C discharge rate grid independence test.

**Fig 5:**
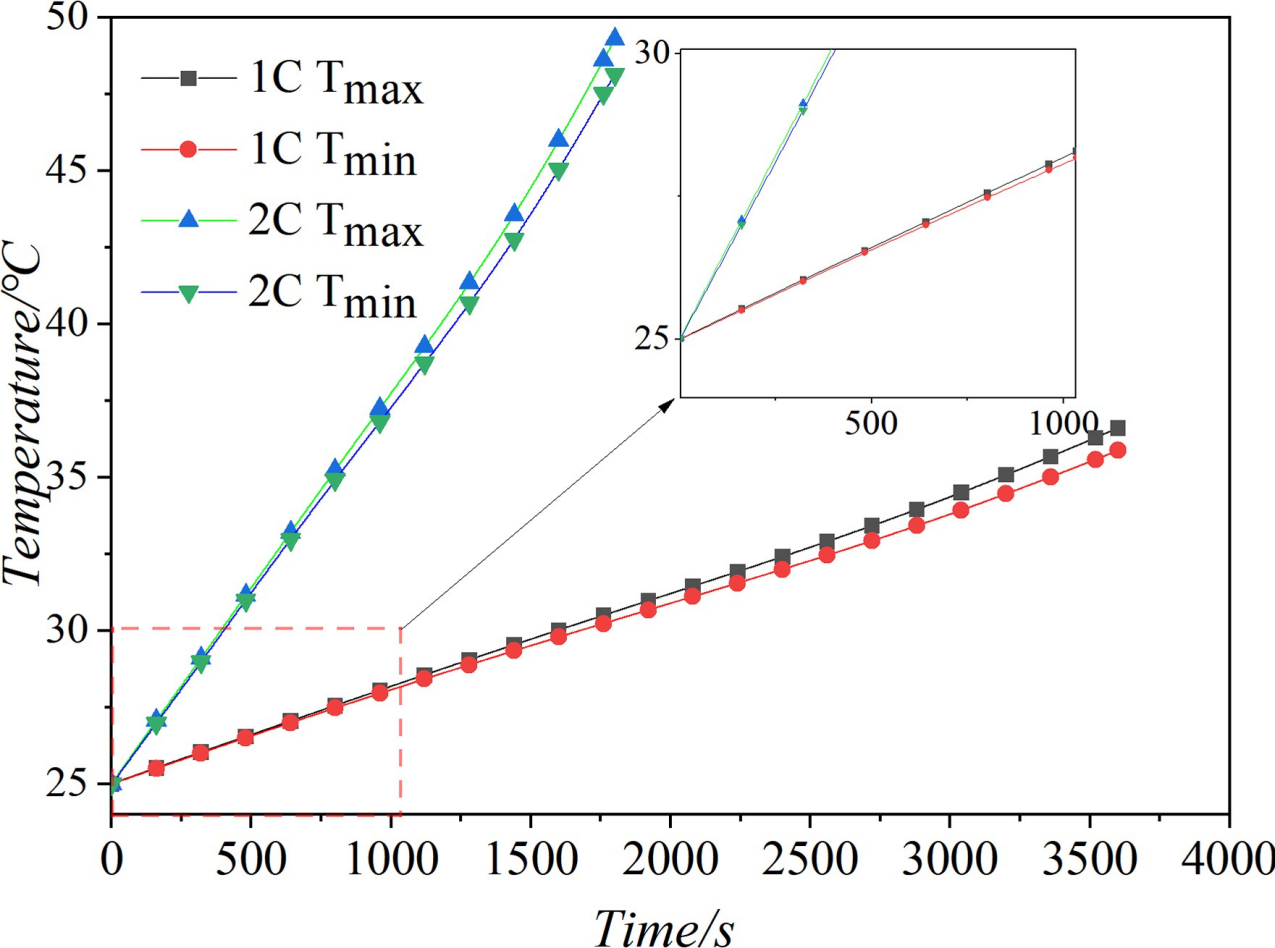
Temperature of battery pack at different discharge rates.

In this study, the battery pack was modeled using *SolidWorks*. A total of 36 square LIBs were arranged in sequence, with a total of 3 battery modules. The LCP is made of aluminum alloy, extruded to form a cold plate channel, and then welded into shape. A 2 mm heat conduction pad was placed between the bottom of the battery pack and the LCP, this pad serves to provide stability against impacts and enhance thermal conductivity. A 0.97 mm epoxy board was placed between the battery cells, primarily serving as insulation to prevent short circuits. The LCP has dimensions of 530*400*3, as depicted in Fig 3. The model is numerically simulated by *Fluent*. To begin with, it is required to determine whether the model interferes and to produce a shared surface. Then, the model was divided using *Fluent meshing*, and a polyhedron mesh was utilized for the model. To determine the appropriate grid number, the mesh independence was verified. The ambient temperature was 25°C, the inlet boundary condition was the velocity inlet, the liquid mass flow rate was 11.29 g/s, the outlet boundary condition was the pressure outlet, and the return water temperature was 25°C. The natural convection heat transfer coefficient of the battery pack was 5 W/(m∙K), and it was discharged at 2C. As depicted in Fig 4, when the number of grids exceeds 4×10^6^, there was a slight variation in the maximum temperature and pressure drop of the battery. Taking into account the impact on simulation time, a similar number of grids will be used to analyze the model. Fig 5 displays the variation of battery pack temperature with time at different discharge rates without any cooling measures. It was found that when the discharge rate was 2C, the temperature of the battery exceeded the optimal operating range Therefore, in this study, the heat dissipation analysis was conducted specifically for the 2C discharge rate.

## 3. Liquid-cooled BTMS design

The study analyzed three battery modules, each containing 12 square shell batteries. The overall battery pack was connected using a 3P12S configuration. Epoxy plates were inserted between the cells to prevent any short circuits between the batteries.

### 3.1. Design of liquid-cooled plate (LCP) structure

**Fig 6:**
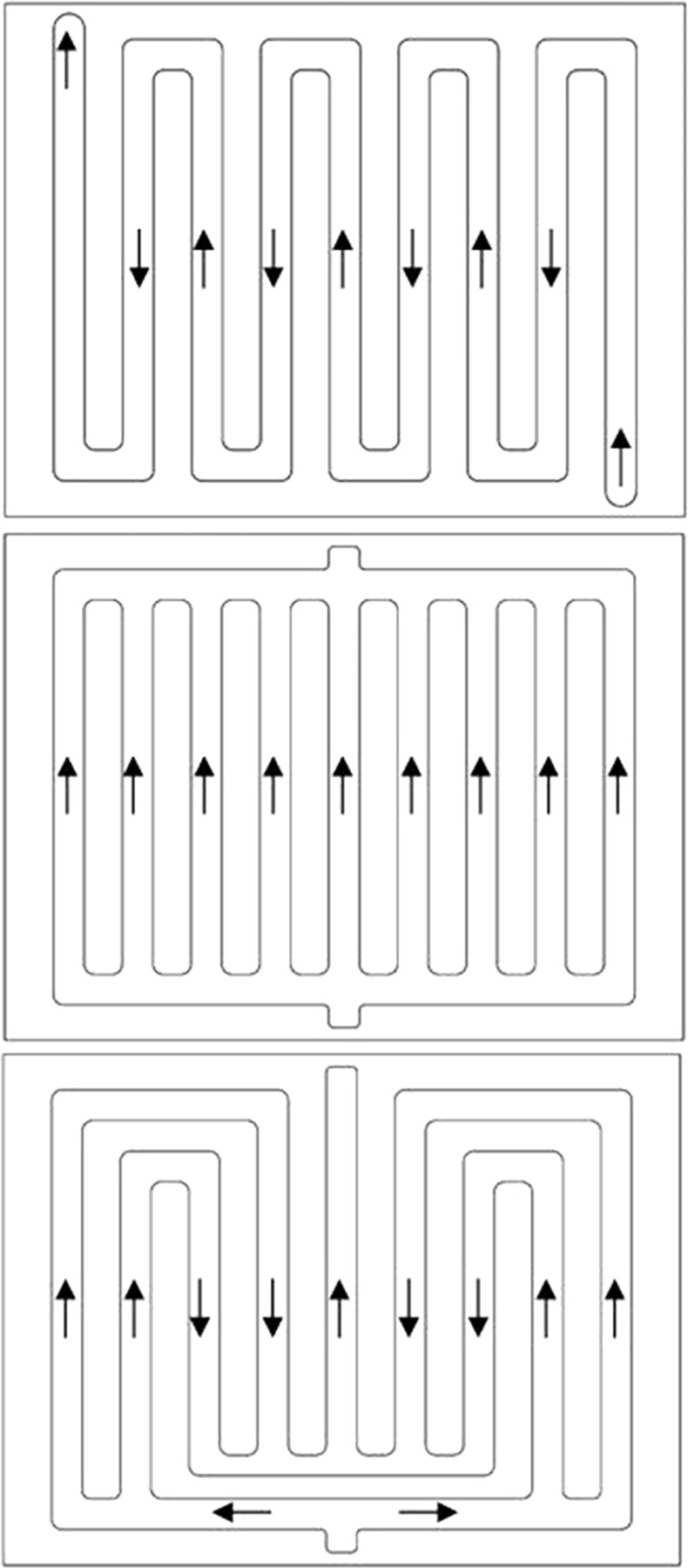
Structural design of LCP with three different flow channels. (a)Serpentine LCP, (b) Parallel LCP, (c) Multi-channel LCP.

The BTMS diagram presented in this article is shown in Fig 3. The thermally conductive silica gel was in contact with the LCP, enabling the dissipation of most of the heat generated by the LIBs. From an economic and energy density perspective, the LCP was constructed from an aluminum alloy, and the runner for the cold plate was extruded. The quantity of cooling channels has a significant effect on the coolant flow path. The adequate arrangement of runners will enhance the heat dissipation efficiency of the battery. As shown in the Fig 6, three different LCPs were used. The depth of the serpentine LCP, parallel channel LCP, and improved multi-channel LCP was all 3 mm, and the width of the coolant channel was 20 mm.

### 3.2. Comparison of LCP structures in different channels

**Fig 7:**
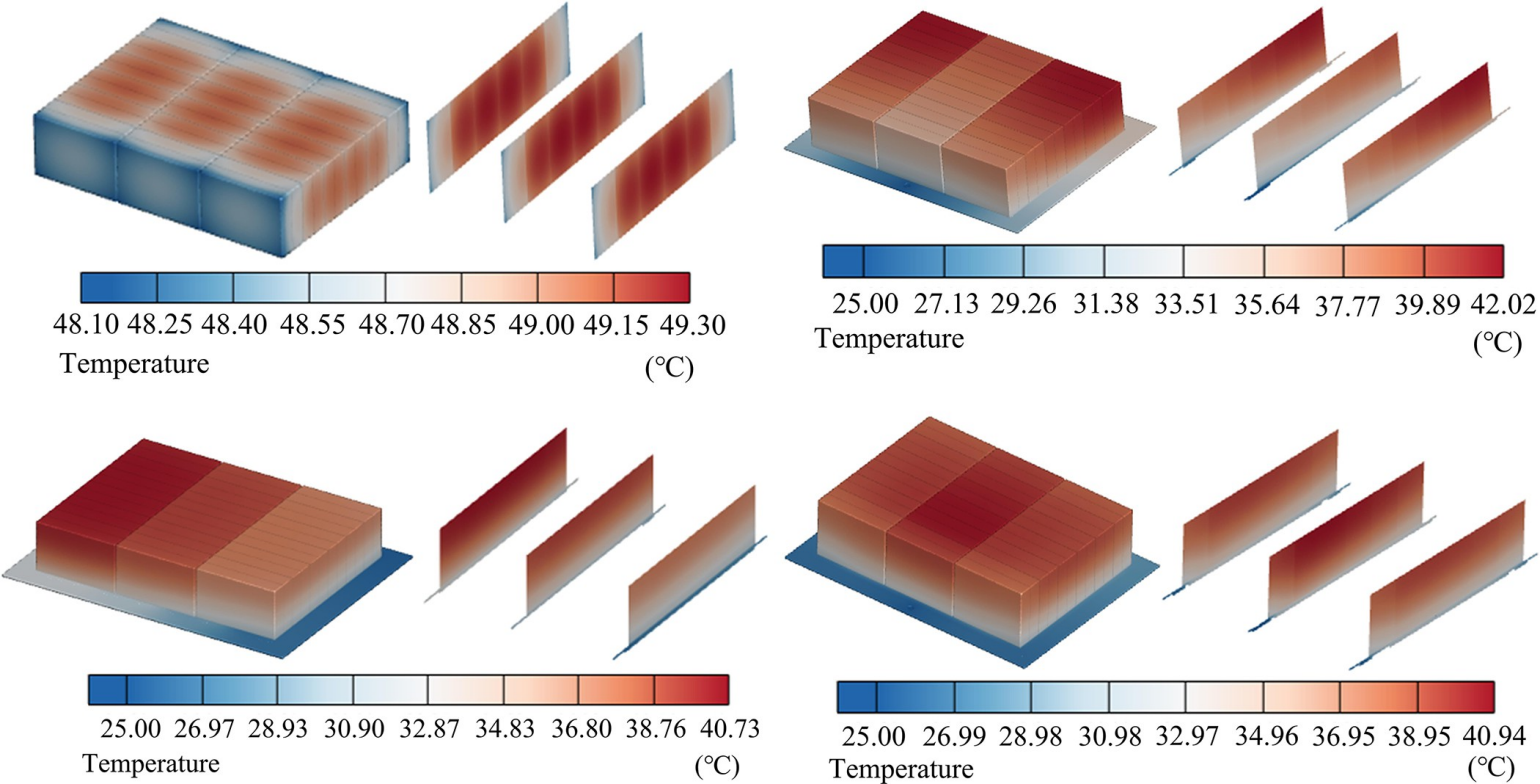
Battery pack and intermediate section temperature of LCP with different flow channels. (a) Non LCP, (b) Serpentine LCP, (c) Parallel LCP, (d) Multi-channel LCP.

**Fig 8:**
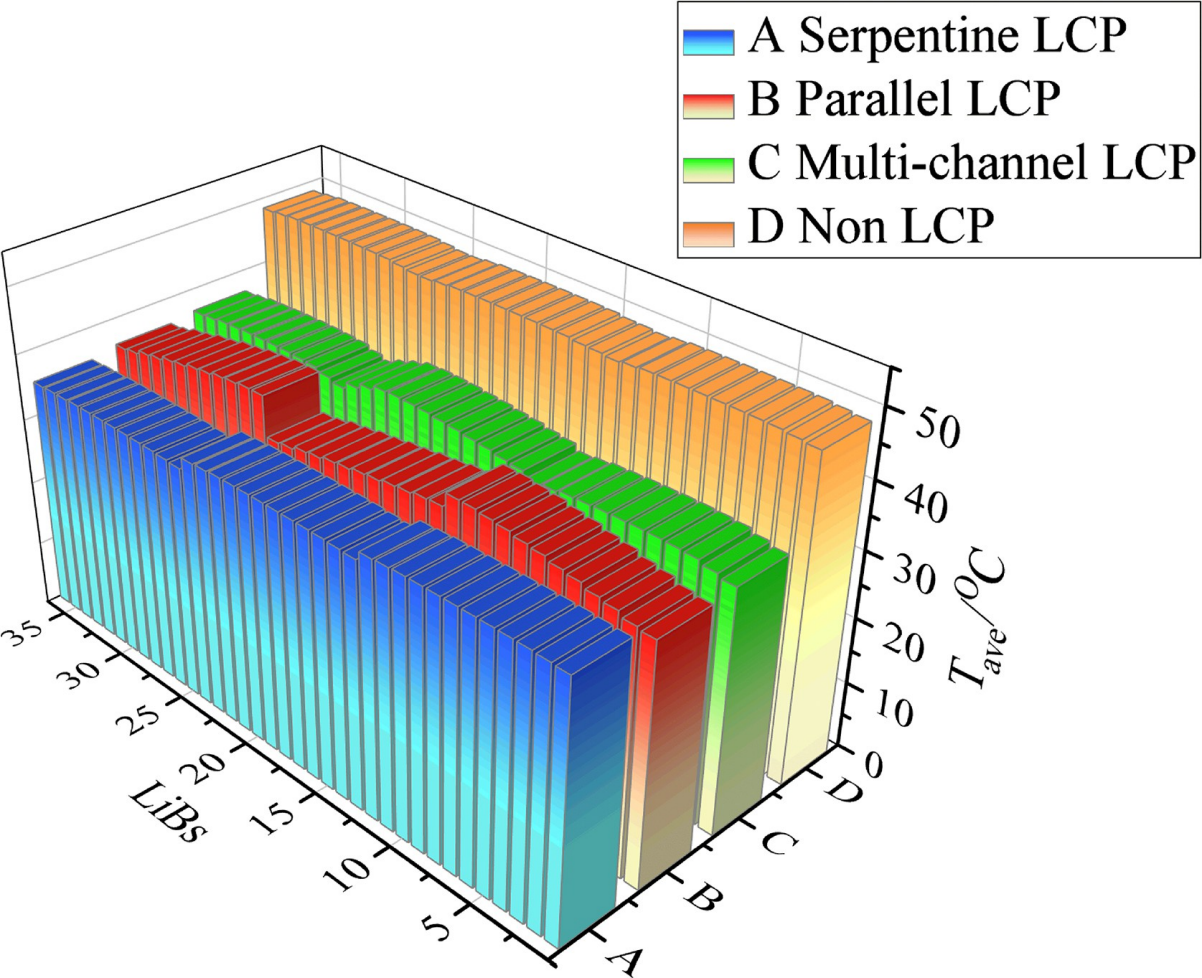
*T*_*ave*_ of 36 batteries with different LCP structures.

The temperature cloud diagram of Lithium-ion Batteries (LIBs) is depicted in Fig 7 after the battery pack has been discharged at 2C, with a coolant mass flow rate of 11.29 g/s. According to the analysis of Fig 7 (A), the maximum temperature (*T*_*max*_) of the battery pack without an LCP is 49.30°C, with a maximum temperature difference (*ΔT*) of 1.20°C. It has been observed that the highest temperature exceeds the normal operating temperature. (b) It is evident that the *T*_*max*_ of the cell in the traditional serpentine LCP is 40.73°C, and the *ΔT* is 11.15°C. (c) The *T*_*max*_ of the cell in the traditional parallel multi-channel LCP is 42.02°C, and *ΔT* is 12.73°C. (d) The *T*_*max*_ of the improved multi-channel LCP is 40.94°C, and *ΔT* is 10.57°C. From Fig 7, it is evident that there is a noticeable concentration of heat at the upper portion of the battery module. The liquid cooling plate is equivalent to the cold source of the whole system and is close to the lower part of the battery pack. By utilizing a heat conduction pad, the LCP is able to rapidly and effectively transfer the heat it generates, and then the cooling liquid dissipates the heat from the system. For three types of liquid cooling systems with different structures, the battery’s heat is absorbed by the coolant, leading to a continuous increase in the coolant temperature. Consequently, it is observed that the overall temperature of the battery pack increases in the direction of the coolant flow. The difference in *T*_*max*_ between the batteries in (d) and (b) is not significant, but the *ΔT* of the battery in (d) is lower than that in (b) and (c). The multi-channel LCP has certain advantages in terms of heat dissipation efficiency of the overall battery pack. The average temperature (*T*_*ave*_) of each battery cell with different LCPs shown in Fig 8, it can be seen that the individual cells *T*_*ave*_ of A and B have a certain degree of variation, while C is relatively stable.

**Fig 9:**
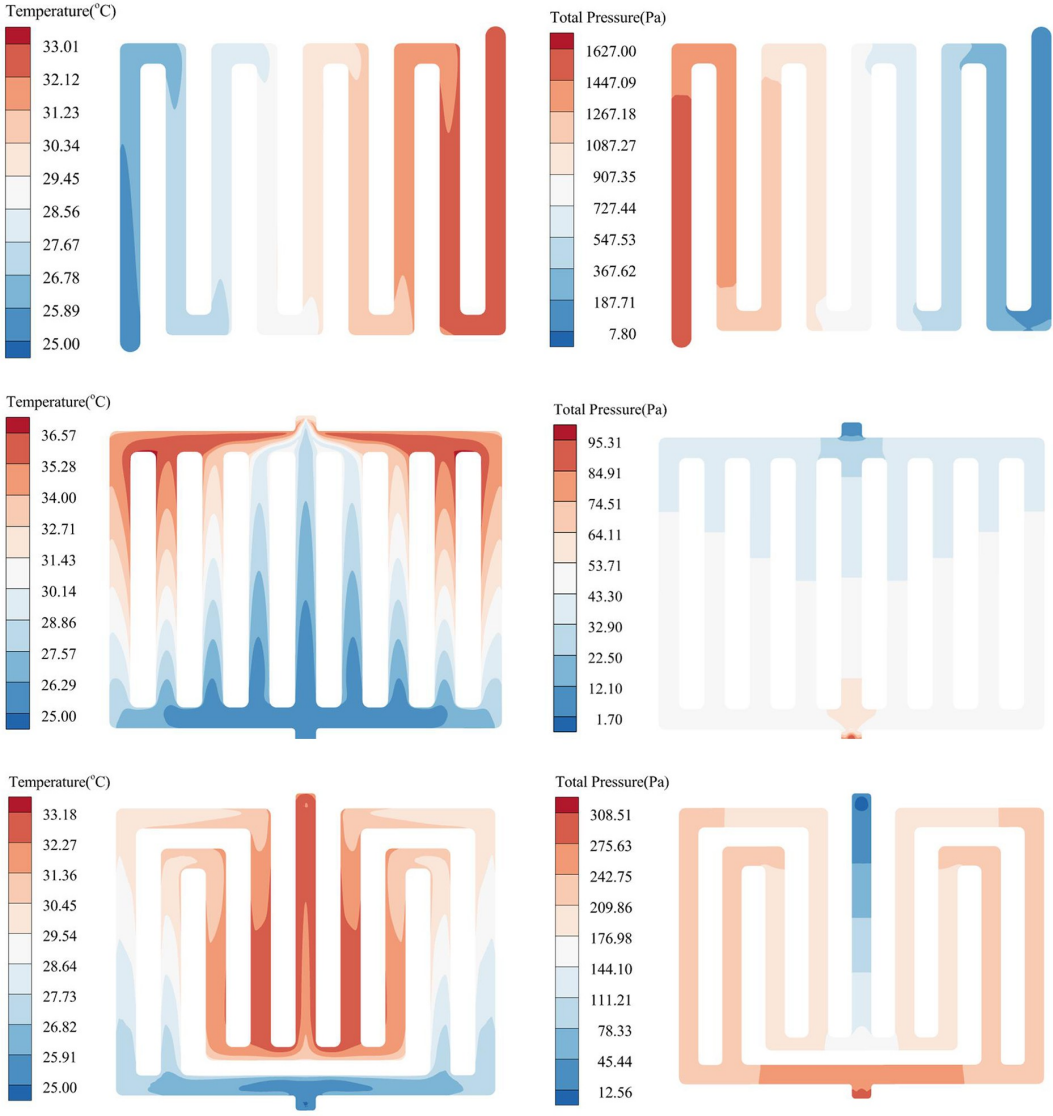
*T* and *ΔP* distribution of coolant in different channel structures. (a) Serpentine-channel coolant *T* and *ΔP*, (b) Parallel-channel coolant T and ΔP, (c) Multi-channel coolant *T* and *ΔP*.

**Fig 10:**
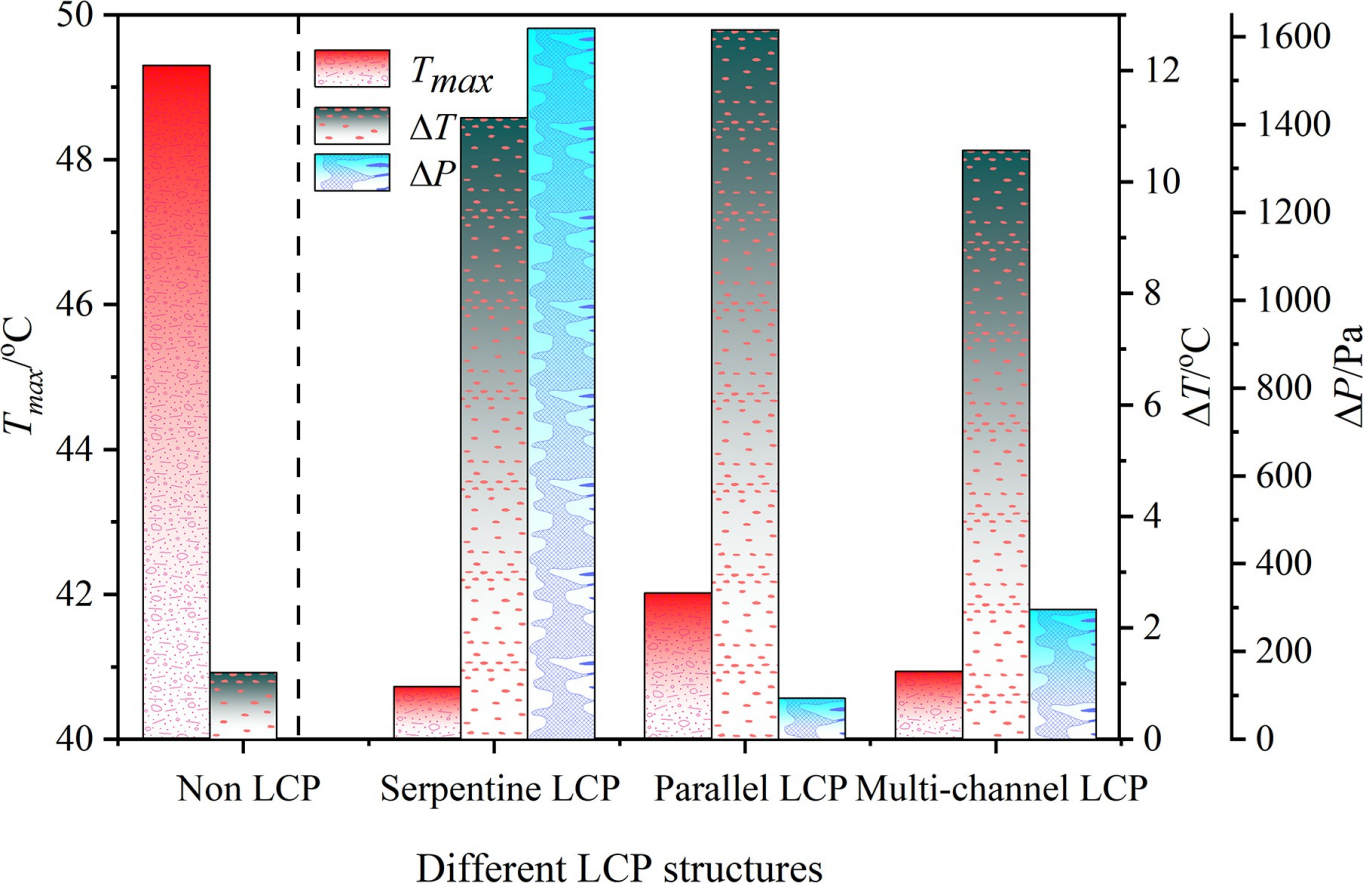
*T*_*max*_, *ΔT* and *ΔP* batteries with different LCP structures.

Additionally, the temperature and pressure drop (*ΔP*) of the coolant for each scheme are shown in Fig 9. (A) The maximum temperature of the coolant is the lowest at 33.01°C, and the pressure drop is the highest at *ΔP* of 1619.20 Pa. (b) The coolant reaches its maximum temperature at 36.57°C, while simultaneously experiencing the smallest pressure drop, with *ΔP* at only 93.61 Pa. The greater the pressure drop, the higher the resistance along the cooling passage, resulting in greater required pump power and ultimately increasing the overall cost of the cooling system. The *T*_*max*_, *ΔT*, and *ΔP* of the battery packs with different LCP structures are depicted in Fig 10. The serpentine structure has a higher coolant speed, leading to a larger system pressure drop, but it offers better cooling performance. The heat from the battery pack is transferred to the coolant in the channel, causing the temperature to gradually increase along the direction of the coolant flow and resulting in a significant temperature difference. The low cooling fluid velocity in the parallel-channel leads to a smaller system pressure drop. Additionally, the absence of auxiliary structures within the channels results in the poorest flow distribution uniformity, with flow velocity in the central channel markedly higher than in the peripheral channels. This is due to the large resistance along the flow path in the outer channel, causing uneven flow distribution and resulting in the highest temperature and temperature difference. In the multi-channel structure, the coolant first flows through the outermost channel. Due to the channel’s symmetry, the flow distribution is uniform, resulting in low pressure drop and smaller maximum temperature and temperature difference. Combined with the thermal management temperature evaluation standard and the system energy consumption standard, the improved multi-channel LCP possesses distinct advantages, *T*_*max*_ is 33.18°C, *ΔP* is 295.95 Pa. Subsequent research and optimization are based on this design.

## 4. Optimal design

**Fig 11:**
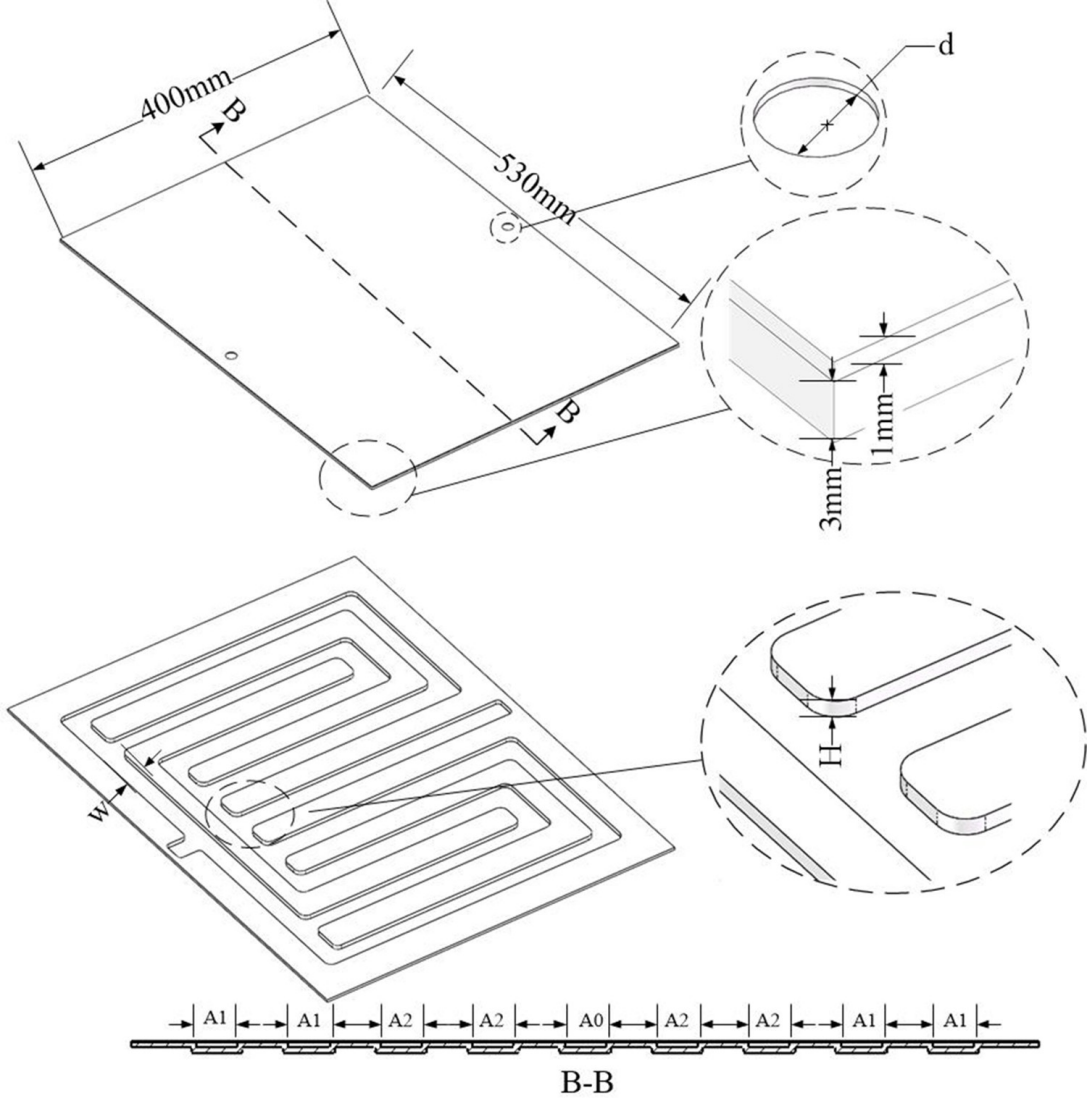
Schematic diagram of LCP structure and flow channel section.

Based on the multi-channel liquid cooling plate mentioned above, the heat dissipation of the battery pack was analyzed, and its structural parameters were optimized. Box-Behnken Design (BBD) [33] experiments were conducted using *Design Expert* software to analyze the impact of channel structure parameters on cooling efficiency. The experiments focused on three channel widths: *A0*, *A1*, and *A2*, as shown in Fig 11. This experiment employs the temperature mean square deviation (*TSD*) of the contact surface between the thermal pad and the LCP to describe the degree of temperature uniformity. The energy consumption of the pump is described by *ΔP*, while the heat dissipation effect is described by *T*_*max*_ of the battery pack. Among them, *d* = 12mm, *w* = 24mm, *H* = 3mm. The range of values for other variables is determined based on the geometric parameter relationship of the LCP, as shown in Table 3. Table 4 displays the arrangement and combination of BBD test factors and response surface values. The temperature mean square deviation is defined as:

$$TSD=\sqrt{\frac{\sum_{i=1}^{n}{\left(T_{i}-\bar{T}\right)}^{2}}{n}}$$ (10)

where *T* is the temperature at node *i* on the examined section, $\bar{T}$ is the average temperature of all examined nodes, and *n* is the total number of examined nodes.

**Table 3:** Design variables and range of values.

design variable	range of values
*A0*	16~32mm
*A1*	16~32mm
*A2*	16~32mm

**Table 4:** Orthogonal experiment scheme and results.

Test Number	Test scheme			Test index		
	*A0*/mm	*A1*/mm	*A2*/mm	*T*_*max*_ (°C)	*TSD*	*ΔP* (Pa)
1	16	24	16	40.974	1.777	397.361
2	16	32	24	40.982	1.812	373.840
3	32	24	32	40.875	1.728	199.239
4	16	24	32	40.977	1.767	352.099
5	24	24	24	40.907	1.740	258.236
6	24	32	16	40.921	1.796	265.137
7	32	24	16	40.855	1.728	221.721
8	24	24	24	40.907	1.740	257.763
9	24	16	16	40.887	1.671	310.304
10	24	24	24	40.906	1.740	258.238
11	16	16	24	40.940	1.684	399.891
12	24	24	24	40.907	1.740	258.236
13	24	24	24	40.907	1.740	258.237
14	32	32	24	40.871	1.768	198.054
15	24	32	32	40.939	1.793	242.528
16	24	16	32	40.890	1.663	268.893
17	32	16	24	40.832	1.642	224.237

### 4.1. Optimization and analysis

Response Surface Optimization (RSM) is distinguished from other data statistical methodologies by combining the evaluation of interactions between independent variables to improve fitting accuracy and the application of graphical techniques to showcase the functional correlation between them. This approach not only enhances accuracy but also facilitates a more intuitive comprehension of the outcomes. The second-order response surface equation employed in the investigation can be formulated as follows [34]:

$$y=\beta_{0}+\sum_{k=1}^{n}\beta_{k}x_{k}+\sum_{k=1}^{n}\beta_{kk}x_{k}^{2}+\sum \sum_{l<k}\beta_{lk}x_{l}x_{k}+\varepsilon$$ (11)

where y is the predicted value of the response surface, *x*_*k*_ is the design variable, *ε* is the residual error, *β*_0_ is the regression constant, *β*_*k*_, *β*_*kk*_ and *β*_*lk*_ are all undetermined coefficients.

The maximum temperature and design variables’ response surface function:

$$\begin{array}{l} T_{max}=41.13-0.012*A0+0.0058*A1-0.0085*A2-1.17e-05*A0*A1+6.64e-05*\\ A0*A2+5.86e-05*A1*A2+8.16e-05*A0^{2}-9.02e-05*A1^{2}-0.00013*A2^{2} \end{array}$$ (12)

Temperature mean square deviation and design variables’ response surface function:

$$\begin{array}{l} TSD=1.594-0.00563*A0+0.02*A1-0.0071*A2-7.81e-06*A0*A1+3.91e-05\\ {}*A0*A2+1.95e-05*A1*A2+4.49e-05*A0^{2}-0.00026*A1^{2}+0.00011*A2^{2} \end{array}$$ (13)

Inlet and outlet pressure drop and design variables’ response surface function:

$$\begin{array}{l} \Delta P=1102.256-35.905*A0-11.174*A1-8.647*A2-0.00052*A0*A1\\ +0.089*A0*A2+0.073*A1*A2+0.48*A0^{2}+0.156*A1^{2}+0.056*A2^{2} \end{array}$$ (14)

**Fig 12:**
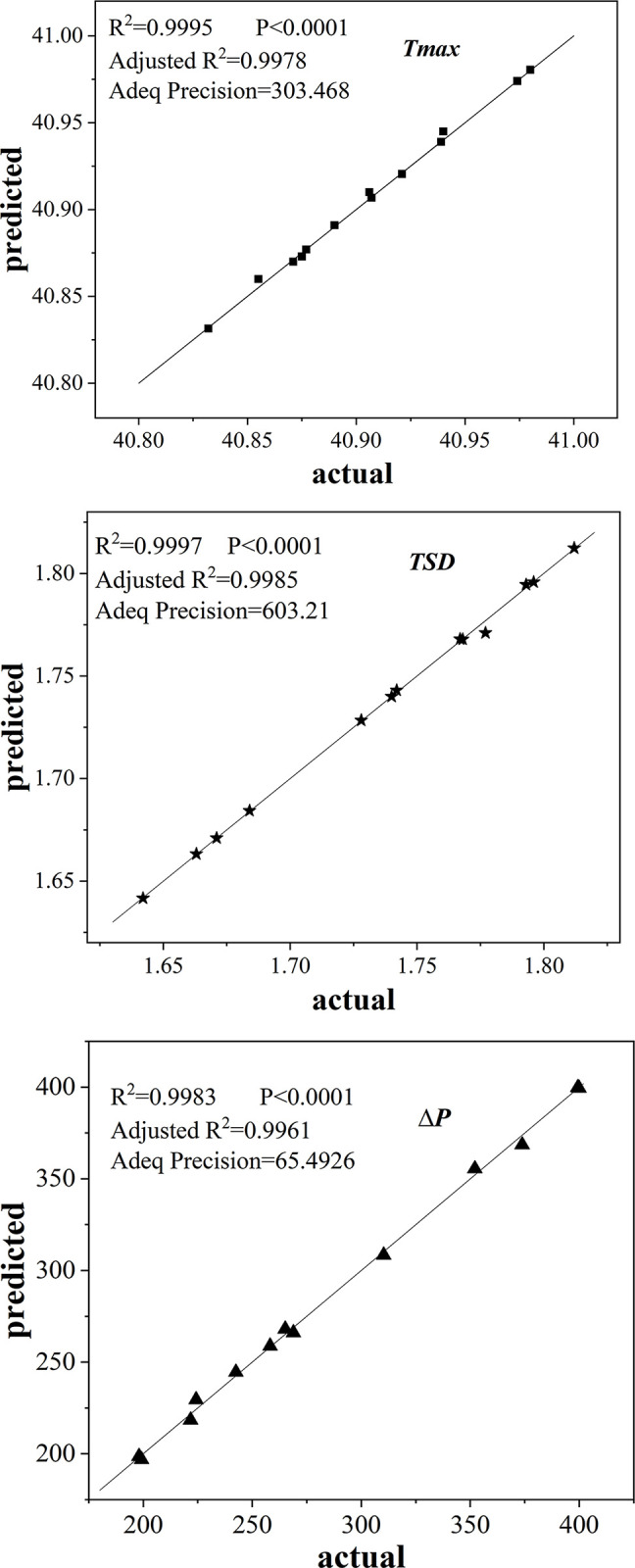
Comparison between actual and predicted values of response surface. (a) Maximum Temperature (b) Temperature Mean Square deviation (c) Inlet and Outlet Pressure Drop.

The comparison of the response surface’s actual and predicted values is shown in Fig 12. It is evident that the predicted values closely align with the actual values, demonstrating negligible deviation. A model’s highly significant influence on the response value is indicated when the model validation value is P<0.0001. The model fits well and the error is minor when the correlation coefficient (R^2^) and correction coefficient (Adj R^2^) both surpass 0.8 and the difference between them is less than 0.2. Furthermore, the model’s resolution index (Adeq Precision) exceeds than 4, indicating a high degree of predictive power. The three models satisfy the requirements based on the results of the analysis of variance performed on the regression equations. Thus, *T*_*max*_, *TSD* and *ΔP* can be analyzed and predicted using the models given in Eqs (12), (13) and (14).

**Fig 13:**
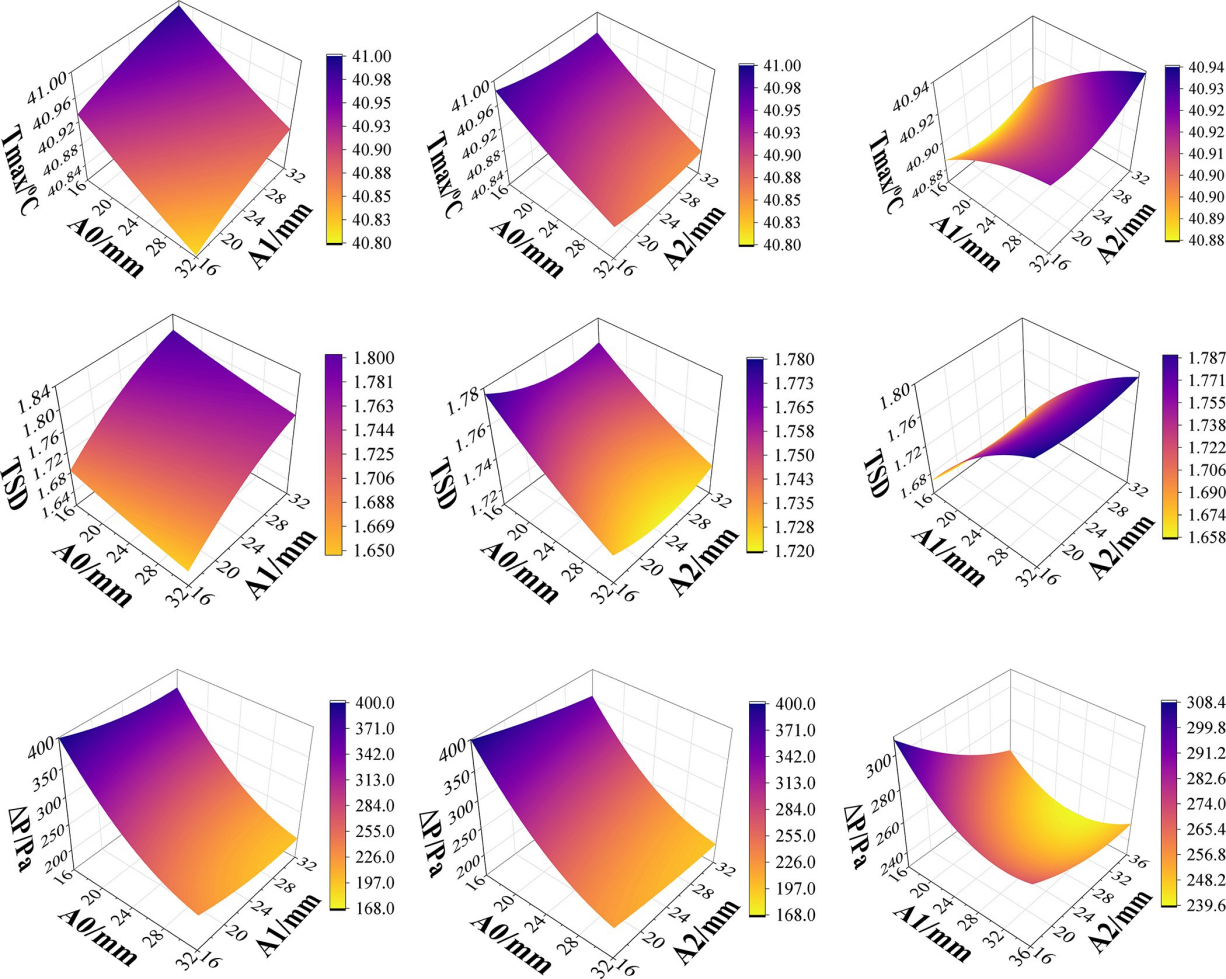
Response surface diagram of channel width parameter. (a) Response surface plot of *T*_*max*_ with respect to *A0*, *A1* and *A2*. (b) Response surface plot of *TSD* with respect to *A0*, *A1* and *A2*. (c) Response surface plot of *ΔP* with respect to *A0*, *A1* and *A2*.

The response surface graph of *T*_*max*_ is obtained from Formula (12). From Fig 13(A), it is clear that *T*_*max*_ falls as *A0* increases when *A2* stays constant, but *A1* shows the opposite trend. Because of the high temperature in the middle of the battery pack, widening *A0* will increase the area in which the coolant and the high-temperature battery come into contact, boosting the battery’s efficiency in dissipating heat. Reducing *A1* is equivalent to reducing the heat carried by the coolant on both sides of the battery pack, thereby improving the heat dissipation efficiency in the middle of the battery pack.

The response surface graph of *TSD* is obtained from Formula (12). From Fig 13(B), it can be seen that when *A0* remains constant, the *TSD* is lowest when *A2* is around 24mm, and the *TSD* decreases as *A1* decreases.

The response surface graph of *ΔP* is obtained from Formula (14). From Fig 13(C), it can be seen that when *A1* remains constant, *ΔP* decreases with the increase of *A0*. When its value reaches the maximum size limit, the pressure drop is minimized, indicating the lowest energy consumption of the system.

According to the above response surface analysis, the maximum temperature, the temperature mean square deviation, and the minimum inlet and outlet pressure drop are the targets, with the target importance *TSD*>*T*_*max*_>*ΔP*. Obtain the optimal solutions *A0* = 32mm, *A1* = 16mm, *A2* = 24.606mm, and obtain *TSD* = 1.642, *ΔP* = 228.365 Pa, and *T*_*max*_ = 40.832°C. Simulated *TSD* = 1.642, *ΔP* = 224.236 Pa, *T*_*max*_ = 40.832°C, The simulation is basically consistent with the predicted value, and it can be seen that *TSD*, *T*_*max*_, and *ΔP* all decrease to a certain extent after optimization.

## 5. DOE experimental design and multi-objective optimization

**Fig 14:**
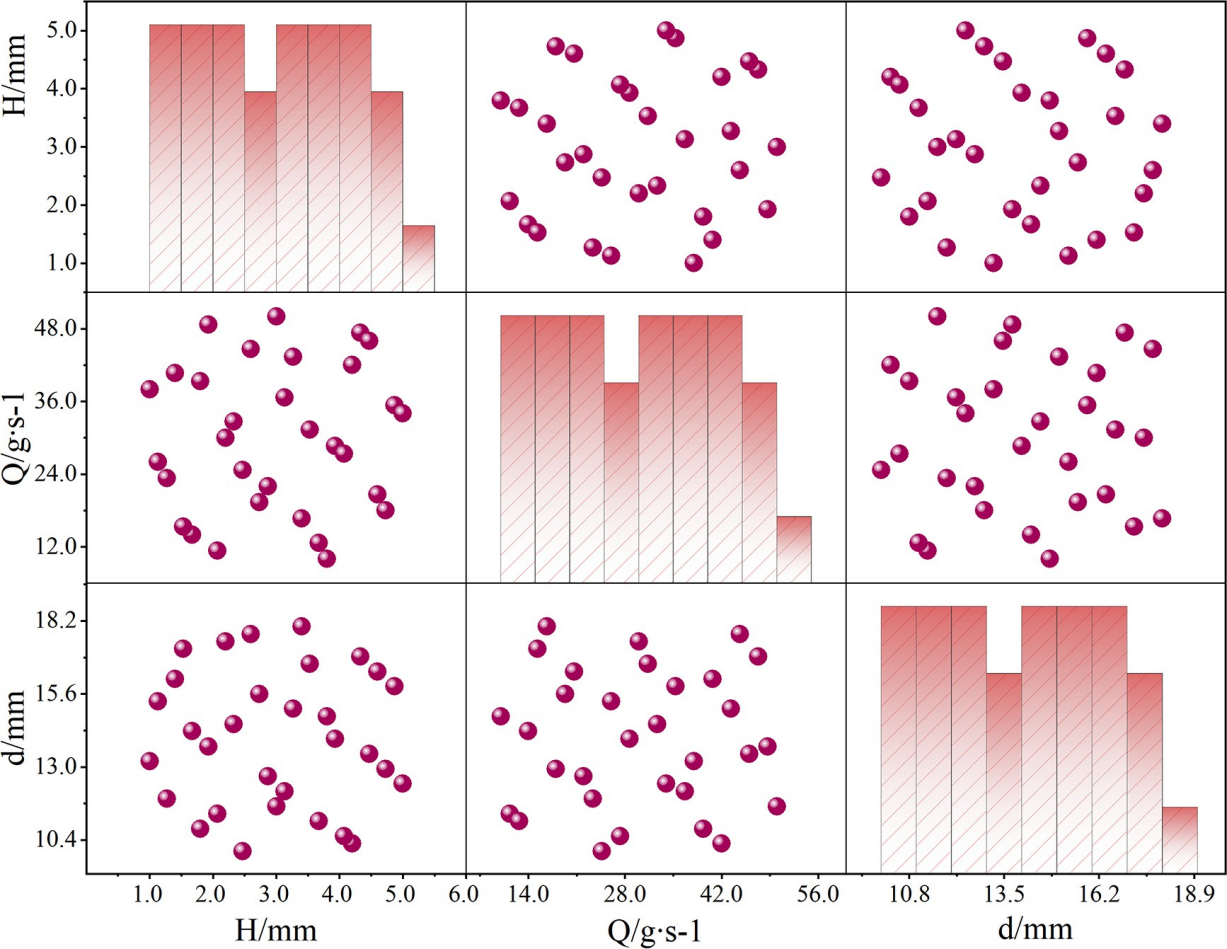
Optimal Latin square sampling distribution.

**Fig 15:**
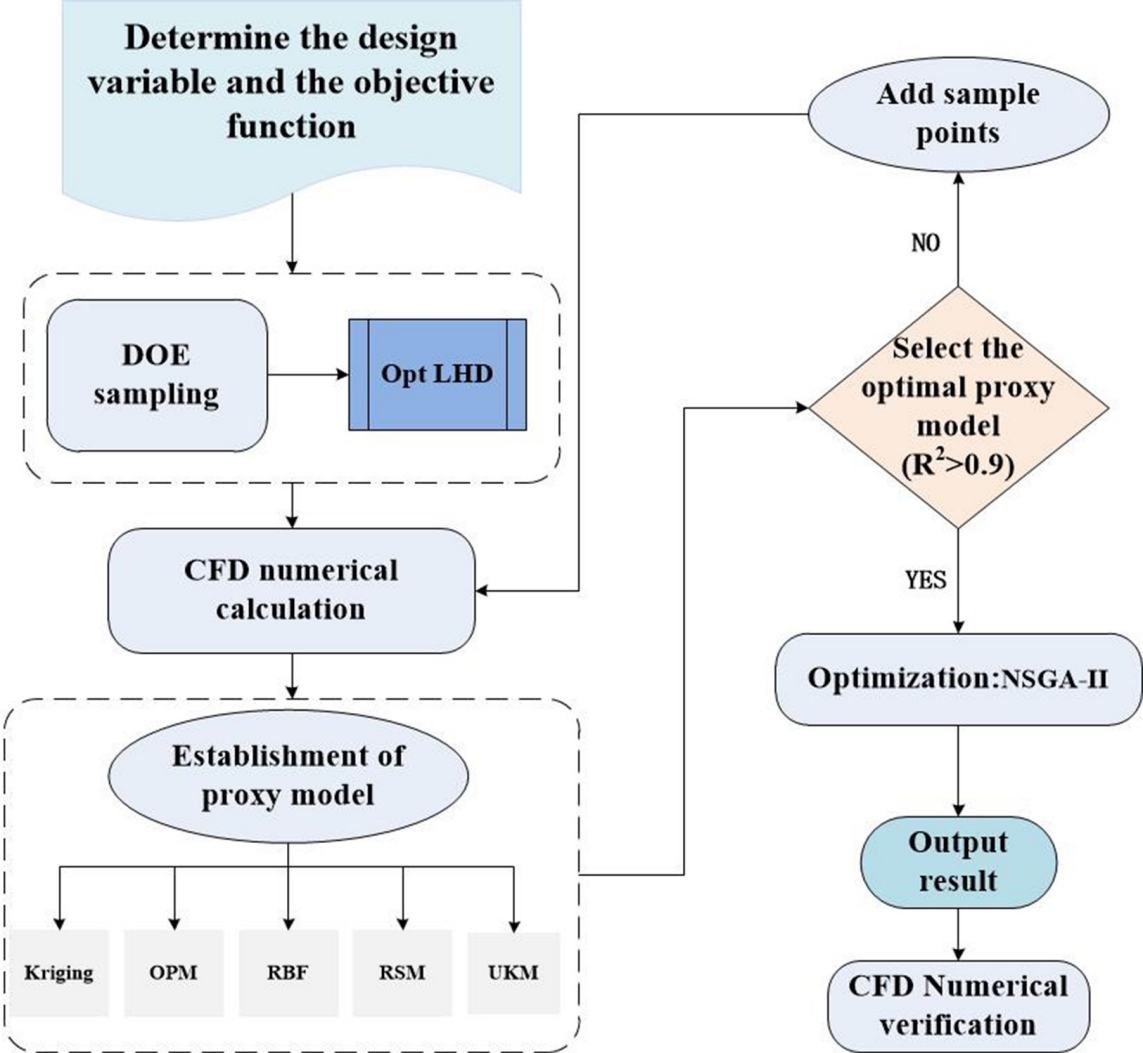
Multi objective optimization design process.

The study obtained sample points through Design of Experiment (DOE) and established a proxy model. The accuracy of the proxy model is directly affected by the number and distribution of these sampling points. To ensure the accuracy of the model, choose an appropriate sampling point acquisition method. The DOE method includes Full Factory Design, Fractional Factory Design, Central Composite Design, Latin Hypercube Design (LHD), Taguchi, etc. Among them, LHD is currently the most widely used method. Although sample points in LHD can be uniformly projected throughout a range of design variables, the random distribution of sample points means that uniform sampling cannot be guaranteed across the whole design space. To make up for the lack of uniformity in LHD, researchers further developed Optimal LHD (Opt LHD). Consequently, as shown in Fig 14, the study sampled the coolant mass flow rate (*Q*), inlet and outlet diameters (*d*), and flow channel depth (*H*) using the Opt LHD approach, yielding 31 sampling points. Fig 15 shows the multi-objective optimization design process.

### 5.1. Selection of proxy models

Sample points were acquired by the Opt LHD method, and the numerical simulation results for each sample point are displayed in Table 5. At present, the methods for establishing proxy models include the Response Surface Model (RSM), Kriging, Radial Basis Functions Model (RBF), Chebyshev/Orthogonal Polynomial Model (OPM), etc. The accuracy of the proxy model was determined by comparing the aforementioned methods, as indicated in Table 6. Consequently, choosing suitable methods to create the ideal proxy model parameters.

**Table 5:** Sample points and numerical simulation results.

#	*H*/mm	Q/g∙s-1	*d/*mm	*T*_max_/°C	*TSD*	Δ*P*/Pa
1	1.27	23.33	11.87	38.77	1.029	6696.82
2	3.27	43.33	15.07	37.81	0.539	1171.96
3	3.93	28.67	14.00	38.46	0.767	422.27
4	2.07	11.33	11.33	40.69	1.691	666.59
5	2.73	19.33	15.60	39.26	1.098	621.71
6	1.40	40.67	16.13	37.81	0.611	9695.76
7	4.60	20.67	16.40	39.14	0.999	173.15
8	4.73	18.00	12.93	39.49	1.092	131.98
9	4.87	35.33	15.87	38.18	0.621	303.19
10	3.80	10.00	14.80	41.15	1.715	107.12
11	3.40	16.67	18.00	39.65	1.216	283.27
12	4.33	47.33	16.93	37.76	0.49	607.03
13	2.47	24.67	10.00	38.71	0.898	1169.29
14	3.67	12.67	11.07	40.42	1.479	168.46
15	1.67	14.00	14.27	40.12	1.480	1590.11
16	1.00	38.00	13.20	37.87	0.687	16786.82
17	4.20	42.00	10.27	37.90	0.542	646.93
18	3.13	36.67	12.13	38.03	0.625	1072.65
19	2.33	32.67	14.53	38.20	0.700	1864.49
20	1.93	48.67	13.73	37.68	0.510	5362.74
21	2.60	44.67	17.73	37.76	0.530	2104.61
22	2.87	22.00	12.67	38.97	0.980	660.93
23	4.07	27.33	10.53	38.57	0.780	368.44
24	3.00	50.00	11.60	37.63	0.480	1848.16
25	1.80	39.33	10.80	37.88	0.610	4952.27
26	1.13	26.00	15.33	38.54	0.940	10178.86
27	5.00	34.00	12.40	38.23	0.640	289.01
28	1.53	15.33	17.20	39.85	1.410	2317.55
29	2.20	30.00	17.47	38.34	0.760	1903.77
30	4.47	46.00	13.47	37.80	0.500	565.72
31	3.53	31.33	16.67	38.29	0.710	611.66

**Table 6:** Comparison of precision of various proxy models.

R^2^	Kriging	OPM	RBF	RSM	UKM
*T*_max_	0.92424	0.98889	0.98034	0.83503	0.99792
*TSD*	0.92427	0.99353	0.98605	0.85006	0.99135
Δ*P*	0.8841	0.85432	0.8501	0.42005	0.90628

Assess each model’s accuracy with the coefficient of determination (*R*^*2*^), which can be expressed as follows [35]:

$$R^{2}=1-\sum_{i=1}^{n}(y_{i}-{\hat{y}}_{i}{)}^{2}/\sum_{i=1}^{n}(y_{i}-{\bar{y}}_{i}{)}^{2}$$ (15)

where *y*_*i*_ represents the actual value, ${\hat{y}}_{i}$ represents the predicted value of the proxy model, ${\bar{y}}_{i}$ represents the average of the actual values, and *n* represents the number of sample points.

**Fig 16:**
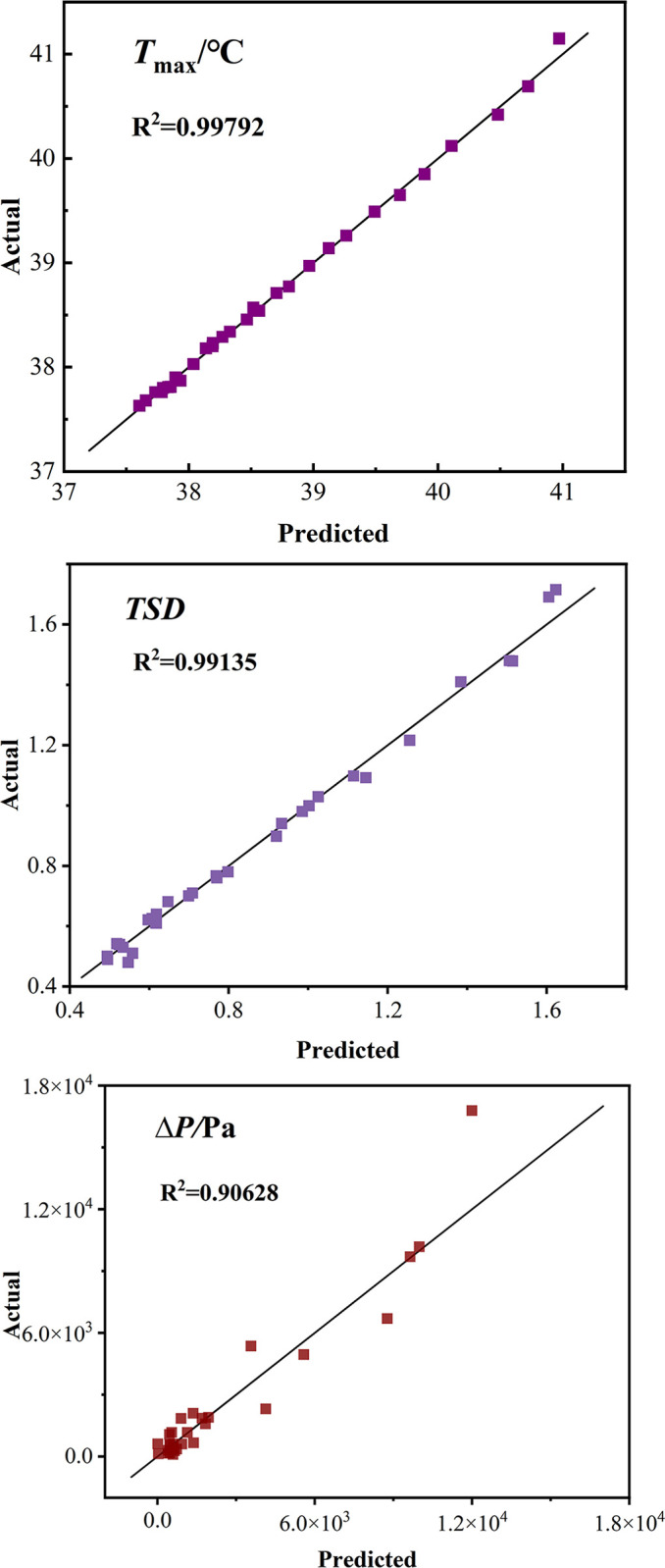
Comparison between actual and predicted values.

The UKM (Universal Kriging Model) is found to have superior accuracy when comparing the *R*^2^ of the proxy models in the above table, and all three indicators’ *R*^2^ values satisfy the accuracy requirements (greater than 0.9). Thus, the UKM serves to serve as a proxy model. The accuracy requirements satisfy with no substantial divergence in comparing the actual and predicted values of *T*_*max*_, *TSD*, and *ΔP* in the UKM, as illustrated in Fig 16. The diagonal represents the predicted values of the UKM, while the data points indicate the actual values.

**Fig 17:**
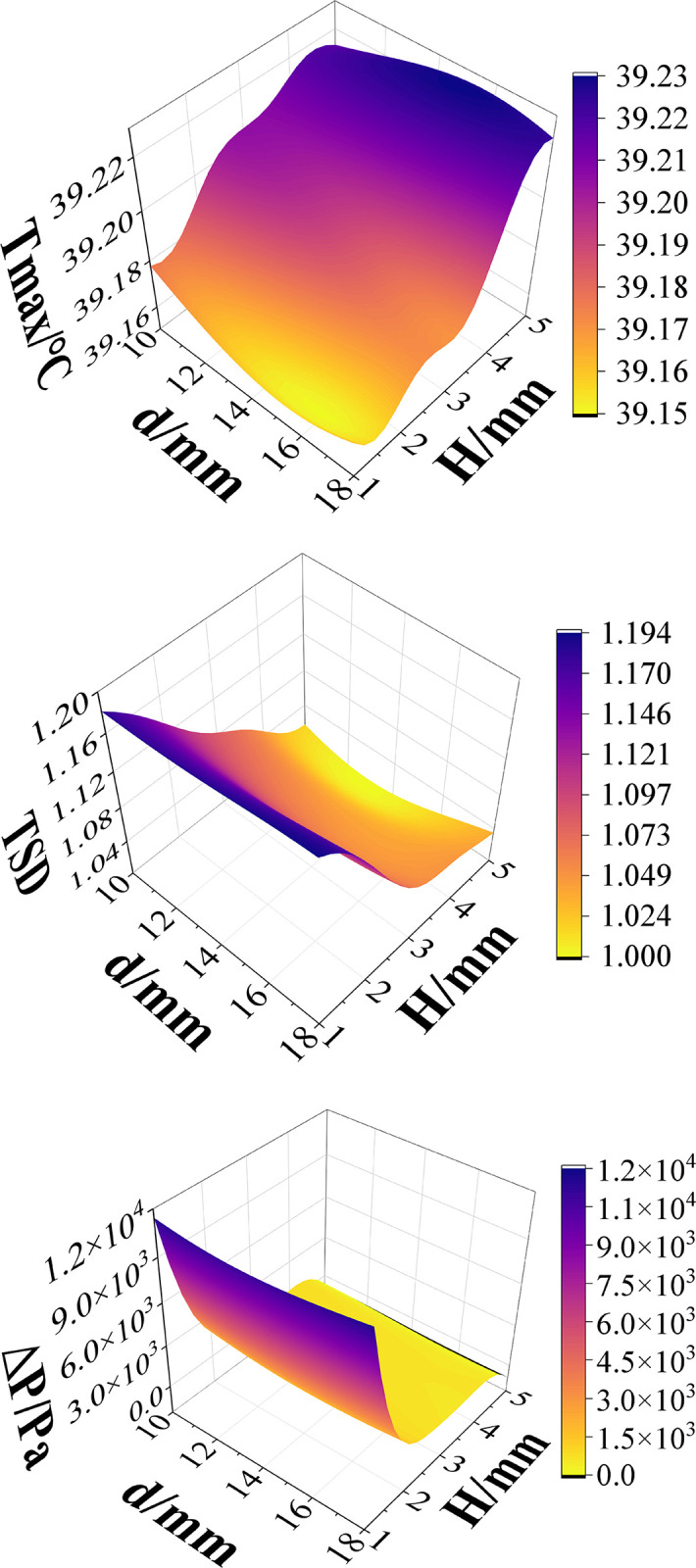
Effect of liquid cooling structural parameters on BTMS at *v* = 0.3m/s.

The influence of different design variables on the objectives can be observed through proxy models. For instance, considering *Q* = 20 g/s, the impact of d and H on these three indicators is depicted in Fig 17. It is observed that maintaining a constant mass flow (*Q*), increasing the diameter (*d*) and decreasing the channel depth (*H*) result in lower maximum battery temperature and reduced surface temperature mean square deviation of the thermal pad, indicating better temperature uniformity. Nevertheless, this causes a rise in pressure drop at the inlet and output, which increases the pump’s energy consumption and power. The pressure drop at the pump’s inlet and output reaches 1.2×10^4^ Pa when it achieves the optimum cooling effect, significantly raising the energy consumption of the pump. To discover the best solution, multi-objective optimization of its structure is therefore required.

### 5.2. Optimization based on multi-objective genetic algorithm

The classical multi-objective genetic algorithm known as NSGA-II uses non-dominated sorting and crowding distance to select and maintain a set of non-inferior solutions. NSGA-II can approach the true Pareto frontier while maintaining the diversity of solutions. This study utilized *Isight* software as the data analysis platform and employed the NSGA-II algorithm on the aforementioned proxy model to identify the optimal solution.

The population size of 16, the genetic algebra of 30, and the hybridization probability of 0.9 were chosen as the primary parameters for the NSGA-II algorithm. And place constraints on the three decision variables, with *TSD*, *T*_*max*_, and *ΔP* serving as optimization goals. *T*_*max*_>*TSD*>*ΔP* is the weight relationship between the three. Establish the target optimization model for the above as follows:

$$\left\{ \begin{array}{l} \mathrm{find}\;x=[H,\;d,\;v]\\ \mathrm{Minimize}\;T_{max}=F_{T}^{*}=(H,\;d,\;v)\\ \;TSD=F_{SDT}^{*}=(H,\;d,\;v)\\ \;\Delta P=F_{P}^{*}=(H,\;d,\;v)\\ s.t.\;1\leq H\leq 5\;10\leq d\leq 18\;0.1\leq v\leq 0.5\\ \mathrm{Weight}\;\mathrm{Factor}\;T_{max}(3)\geq TSD(2)\geq \Delta P(1) \end{array}\right.$$ (16)

**Fig 18:**
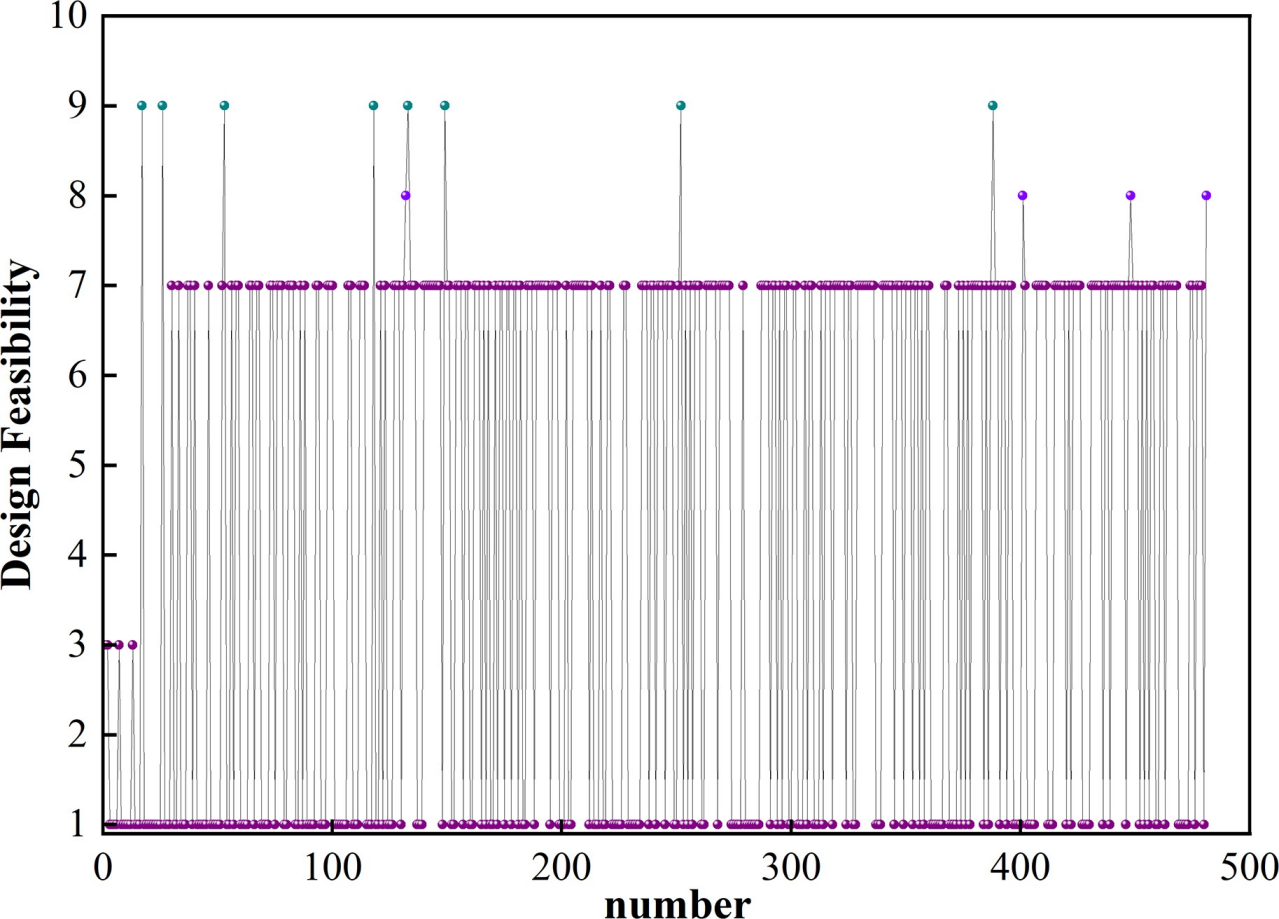
Feasibility diagram of design points.

The NSGA-II algorithm produced 480 sets of feasible design point data, as illustrated in Fig 18. The value of Design Feasibility (DF) ranges from 1 to 9, and the closer it is to 9, the closer the solution of the design point is to the optimal solution. To satisfy the goal optimization requirements, the value of DF should generally be more than or equal to 7.

The Pareto solution set constructed up of design points with DF values greater than or equal to 7. The mean square deviation of the thermal pad’s surface temperature, the battery’s maximum temperature, and the pump’s power consumption are all negatively correlated. The reduction in maximum temperature and temperature mean square deviation comes at the cost of an increase in power consumption. The optimal values for each design objective cannot be achieved simultaneously, a set of DF values of 9 is taken from Pareto as the optimal solution. Therefore, substituting *d* = 14.82mm, *H* = 4.76mm, and *Q* = 35.12g/s into the simulation model to calculate, and Table 7 shows a comparison to its initial structure. The simulation is basically consistent with the data obtained based on the proxy model and NSGA-II, which proves its accuracy.

**Table 7:** Comparison between initial structure and optimized structure.

	Design variable			Optimization goal		
	*d*/mm	*H*/mm	Q/g∙s^-1^	*T*_max_/°C	*TSD*	Δ*P*/kPa
Initial structure	12	3	11.29	40.94	1.69	0.296
NSGA-II	14.82	4.76	35.12	38.12	0.63	0.254
CFD simulation	14.82	4.76	35.12	38.14	0.64	0.291

### 5.3. Heat dissipation effect of optimized model

**Fig 19:**
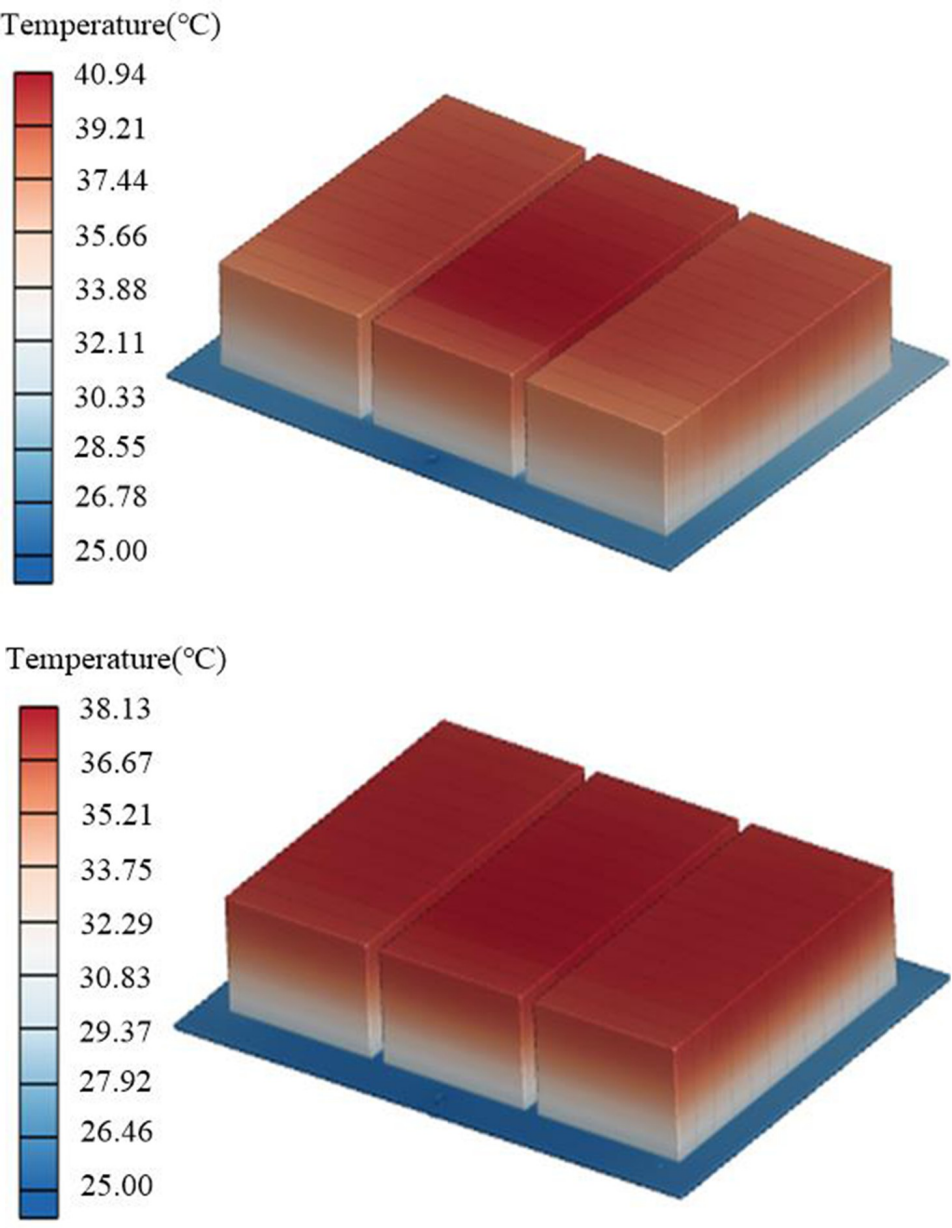
Temperature comparison of battery modules before and after optimization. (a) Initial battery pack temperature, (b) Optimized battery pack temperature.

**Fig 20:**
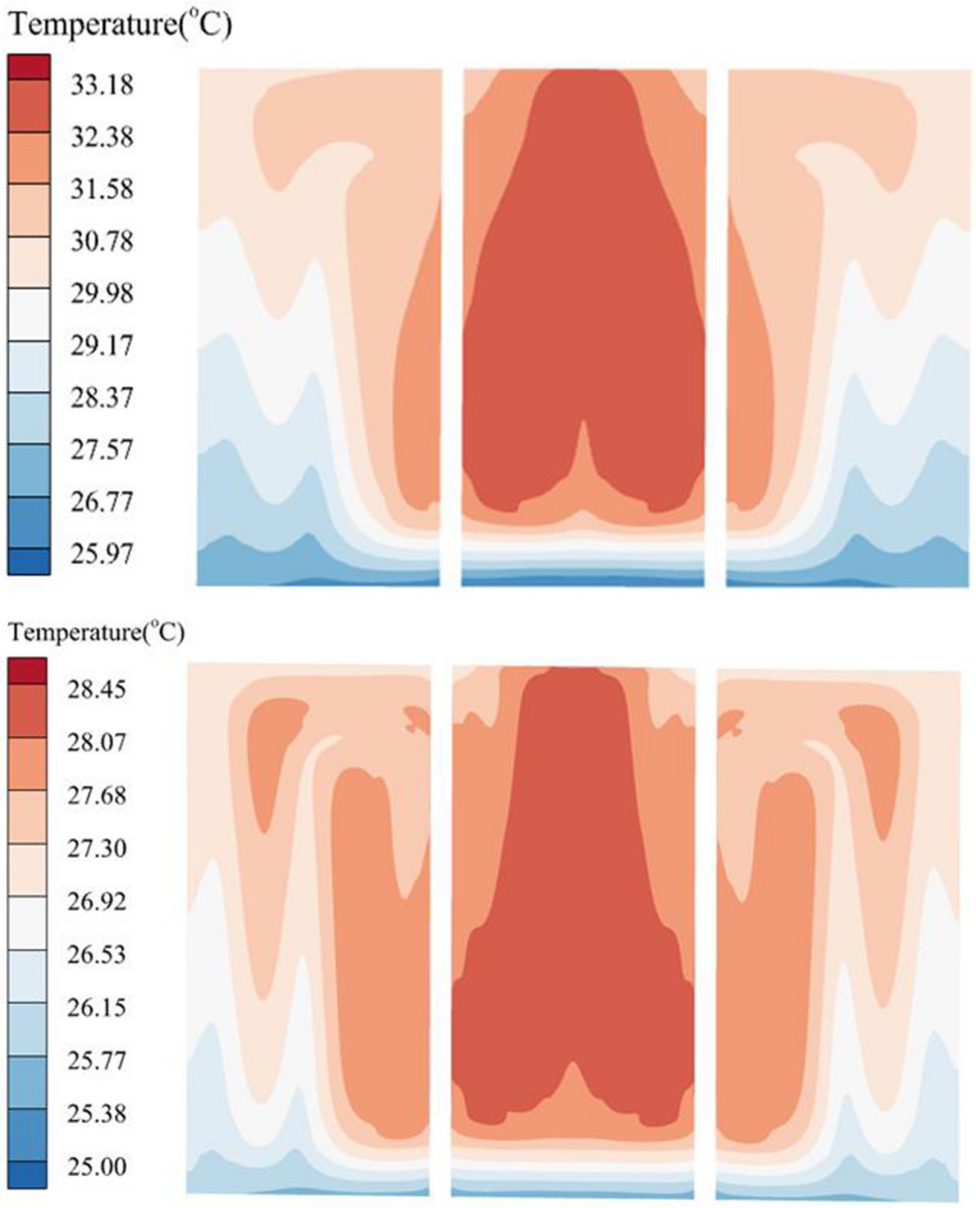
Surface temperature of thermal conductive pads before and after optimization. (a) Initial surface temperature of thermal conductivity pad, (b) Optimized surface temperature of thermal conductivity pad.

From the comparison between the initial structure and the optimized structure shown in Table 7, it can be seen that the *T*_*max*_ of the battery module corresponding to the optimized structure decreased from 40.94°C to 38.14°C, a decrease of 6.84%; the *TSD* decreased from 1.69 to 0.64, a decrease of 62.13%; the *ΔP* of the coolant inlet and outlet increased from 0.296 kPa to 0.291 kPa, with little difference between the two. It is evident that the heat dissipation performance of the improved structural battery module has greatly increased. While meeting the heat dissipation requirements, Δ*P* has not significantly increased, and the pump can achieve the heat dissipation target with lower power consumption. Fig 19 shows that the *T*_*max*_ of the initial structure battery pack is mainly distributed on the middle upper surface, while the optimized temperature distribution of the battery pack is relatively uniform, with some improvement in *T*_*max*_ and Δ*T*. Fig 20 shows the temperature cloud map of the contact surface between the thermal conductive pad and the LCP. The maximum temperature difference on the surface of the initial structure thermal pad is 7.21°C, and the temperature of the middle surface is excessively high, causing the battery’s highest temperature to be excessively high. After optimization, the maximum temperature difference of the contact surface is only 3.45°C, the *TSD* is decreased, and the overall heat dissipation effect is improved.

**Fig 21:**
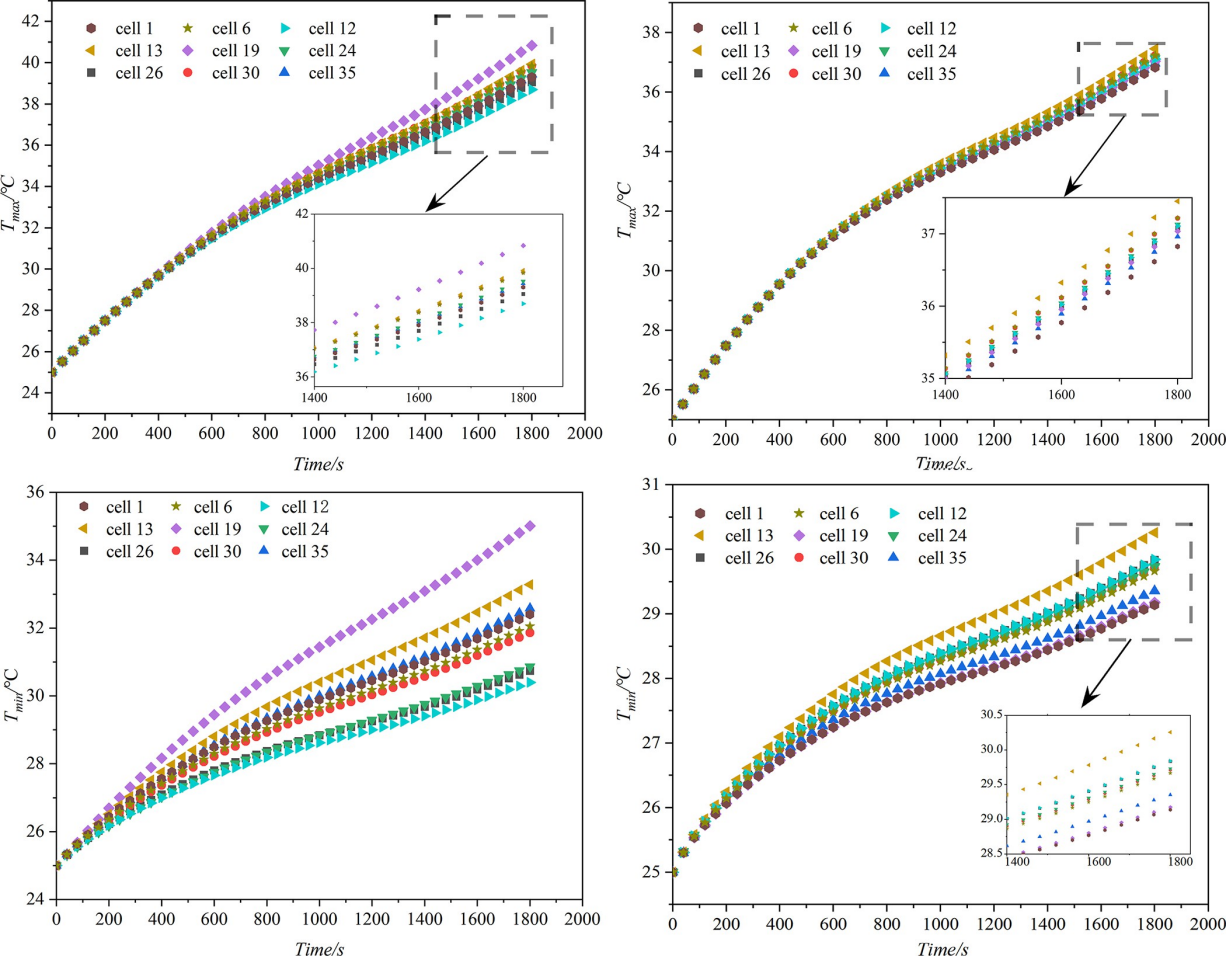
Initial structure and optimized structure Battery *T*_*max*_ and *T*_*min*_. (a) *T*_*max*_ of the initial part of the battery, (b) *T*_*min*_ of the initial part of the battery, (c) *T*_*max*_ of the optimized part of the battery, (d) *T*_*min*_ of the optimized part of the battery.

The maximum and minimum temperatures of part batteries in the initial structure are depicted in Fig 21(A) and 21(B), respectively. The maximum difference in *T*_*max*_ between different batteries is close to 2°C, and the maximum difference in *T*_*min*_ is close to 5°C. The overall temperature difference is relatively large, resulting in higher local temperatures that impact the battery’s performance. The greatest and lowest temperatures of part batteries following optimization are shown in Fig 21(C) and 21(D). The maximum difference in *T*_*max*_ between different batteries is less than 1°C, and the maximum difference in *T*_*min*_ is less than 1.5°C. Therefore, the liquid cooling system’s overall battery heat dissipation efficiency has somewhat increased.

## 6. Conclusion

This study proposes three types of microchannel liquid-cooled plates and evaluates their heat dissipation and energy consumption through Computational Fluid Dynamics (CFD) analysis. The multi-channel liquid cooling plate features efficient heat dissipation and low energy consumption. Box-Behnken experimental design was adopted to determine the channel width parameters. Finally, using the flow channel depth, coolant mass flow rate, and inlet and outlet diameters as design parameters, the optimal Latin square was employed to sample each parameter. A proxy model was then established to analyze the influence of these design parameters on the response target. And multi-objective optimization was carried out with NSGA-Ⅱ.

(1) The *T*_*max*_ of the battery pack with multi-channel liquid cooling plates is 40.94°C, and the required power *ΔP* is 295.95 Pa.

(2) The optimization was carried out through response surface experiments. The temperature mean square deviation, maximum temperature, and pressure drop all decreased to a certain extent, with *TSD* = 1.642, *ΔP* = 224.236 Pa, and *T*_*max*_ = 40.832°C。

(3) Through multi-objective optimization of design parameters, The *T*_*max*_ decreased from 40.94°C to 38.14°C, a decrease of 6.84%; The temperature mean square deviation (*TSD*) decreased from 1.69 to 0.63, a decrease of 62.13%; The optimized structural battery module has significantly improved heat dissipation performance.

In future work, research will be conducted on heating battery packs in low-temperature environments and further research will be conducted on other novel optimization methods, such as topology optimization.

## Abbreviations

*A0*, *A1*, *A3*: channel widths (mm)
*C*: specific heat capacity (J/(kg k))
*d*: diameters (mm)
*H*: flow channel depth (mm)
*I*: current (A)
*n*: the number of sample points
*P*: Pressure (Pa)
*ΔP*: pressure drop (Pa)
*q*: heat production rate (W/m^3^)
*Q*: mass flow rate (g∙s-1)
*R*: Resistance (mΩ)
*Re*: Reynolds number
*R^2^*: the coefficient of determination
*T*: temperature (°C)
*t*: time (s)
*U*: voltage (V)
*v*: velocity (m/s)
*V*: volume of the battery, m3
*x, y, z*: the coordinate axis
*y_i_*: actual value
${\hat{y}}_{i}$: predicted value
${\bar{y}}_{i}$: the average of the actual values
*Greek symbols ρ*: density (kg/m3)
*λ*: heat coefficient (W/(m·K))
*μ*: dynamic viscosity (Pa s)
*ε*: residual error
*Subscripts* 0: Initial value
*ave*: average
*l*: liquid-cooled plate
*max*: maximum
*min*: minimum
*w*: coolant
*Acronyms* BBD: Box-Behnken Design
BTMS: Battery Thermal Management System
DOE: Design Of Experiments
LCP: Liquid Cooled Plates
LHD: Latin Hypercube Design
LIBs: Lithium-ion Batteries
RSM: Response Surface Model
TSD: Temperature mean square deviation
UKM: Universal Kriging Model
